# The impact of genome variation and diet on the metabolic phenotype and microbiome composition of *Drosophila melanogaster*

**DOI:** 10.1038/s41598-018-24542-5

**Published:** 2018-04-18

**Authors:** Lisa Jehrke, Fiona A. Stewart, Andrea Droste, Mathias Beller

**Affiliations:** 1 Institute for Mathematical Modeling of Biological Systems, Heinrich Heine University, Duesseldorf, Germany; 2 Systems Biology of Lipid Metabolism, Heinrich Heine University, Duesseldorf, Germany

## Abstract

The metabolic phenotype of an organism depends on a complex regulatory network, which integrates the plethora of intrinsic and external information and prioritizes the flow of nutrients accordingly. Given the rise of metabolic disorders including obesity, a detailed understanding of this regulatory network is in urgent need. Yet, our level of understanding is far from completeness and complicated by the discovery of additional layers in metabolic regulation, such as the impact of the microbial community present in the gut on the hosts’ energy storage levels. Here, we investigate the interplay between genome variation, diet and the gut microbiome in the shaping of a metabolic phenotype. For this purpose, we reared a set of fully sequenced wild type *Drosophila melanogaster* flies under basal and nutritionally challenged conditions and performed metabolic and microbiome profiling experiments. Our results introduce the fly as a model system to investigate the impact of genome variation on the metabolic response to diet alterations and reveal candidate single nucleotide polymorphisms associated with different metabolic traits, as well as metabolite-metabolite and metabolite-microbe correlations. Intriguingly, the dietary changes affected the microbiome composition less than anticipated. These results challenge the current view of a rapidly changing microbiome in response to environmental fluctuations.

## Introduction

The metabolic phenotype of an organism depends on a multitude of internal and external parameters such as the genetic repertoire, stress levels, immune system activity, gut microbiome composition and the quality and caloric content of the diet. The orchestrated response to all of these cues ultimately shapes a given metabolic phenotype, characterized by a distinct combination of metabolic parameters. Of those, the relative distribution of energy rich storage compounds, as well as their absolute amounts, are of particular interest. Depending on the context, a given metabolic phenotype may be advantageous or deleterious. High lipid storage levels, for example, are advantageous in times of elevated energy consumption or limited food availability. For many humans, however, modern lifestyle involves an overflow of high caloric food and too little physical activity, which results in lipid storage levels high enough to provoke many detrimental consequences and ultimately decrease total lifespan^1^. On top of lifestyle effects, genome-wide association studies identified numerous obesity-related genome variants in different natural populations^2–4^ demonstrating that physiology regulation is additionally under genetic control. Given the surge of metabolic diseases, a better understanding of the underlying regulatory network causing a certain metabolic phenotype is mandatory. Unfortunately, however, results from e.g. the genome-wide association studies depicted that metabolic regulation is multigenic and multifactorial due to the impact of varying environmental effectors. This complexity results in a very limited understanding of the principles governing organismic resource allocation and the regulatory network shaping a given metabolic phenotype. The identification of further layers of regulation additionally hampers our understanding. For example, research over the last decade involving human epidemiological data^5^ but also targeted experiments using e.g. the fruit fly^6^ or mice^7^, revealed that a process described as “paternal metabolic programming” transmits the dietary preferences of fathers to their offspring via epigenetic imprinting and causes e.g. an increased probability to develop an obese phenotype. Another more recently discovered factor that influences the metabolic phenotype is the gut microbiome^8–10^. All multicellular organisms live in symbiosis with vast amounts of prokaryotes, which influence almost every aspect of the host’s physiology^11^. The bacteria provide or facilitate access of the host to a vast variety of metabolites including nutrients such as vitamins or essential amino acids^12,13^ or increase the possible energy harvest from the diet^14,15^. More importantly, however, the microbiome also contributes to regulate nutrient allocation^16,17^. The gut microbiome of *Drosophila* is much simpler as compared to mammals and mainly consists of only five operational taxonomic units (OTUs; *Acetobacter pomorum*, *A*. *tropicalis*, *Lactobacillus brevis*, *L*. *fructivorans* and *L*. *plantarum*) that vary in their abundance during development and aging of the fly^18^. The reduced complexity of the *Drosophila* microbiome vastly facilitates the analysis of microbiome – metabolism interactions.

To gain better insights into the intricate metabolic regulatory network shaping a given metabolic phenotype we used *Drosophila melanogaster* as a model organism. The *Drosophila melanogaster* Genetic Reference Panel (DGRP) is an ideal resource to study the effects of natural genomic variation under different environmental conditions and consists of approximately 200 wild-derived inbred fly lines that are fully sequenced. A subset of about 40 lines from the DGRP covers a large portion of the phenotypic space^19,20^, thus facilitating profiling experiments. In order to learn more about the interplay between genome variation, the gut microbiome and environmental conditions, we reared the core-set of DGRP flies under basal and nutritionally challenged conditions and subjected the animals to metabolic and microbiome profiling experiments. Across the fly lines and experimental conditions, we performed absolute quantifications of different key metabolites such as triacylglycerols (TAG), glucose, glycogen and lactate. Hierarchical clustering of the fly lines based on the metabolic data revealed the presence of cohorts of fly lines sharing a characteristic metabolite profile, which we termed “metabotype”. Principal component analysis revealed that the limited number of metabolites quantified by us were already sufficient to separate reliably larval, female and male samples. The only exception were four DGRP fly lines where female flies showed a male-like metabolite profile. In subsequent fecundity experiments, females of two of these fly lines indeed showed a reduced egg-laying rate, suggesting that the male-like metabolite profile is indicative of a masculinization. In order to learn more about the coupling of metabolic pathways we performed correlation analyses. Our results suggest an intimate coupling between contextual metabolite abundancies and revealed so far unexpected, highly significant co-abundancies, which help to distinguish different cohorts of metabolically related fly lines. Our microbiome sequencing results obtained for selected DGRP fly lines reared under standard and metabolically challenged conditions challenge the current view that the *Drosophila* microbiome composition rapidly changes in response to environmental fluctuations, as we did not recognize prominent changes in response to the diet changes we performed.

## Materials and Methods

### *Drosophila* stocks and fly rearing

The fly lines covered a subset of the *Drosophila melanogaster* Genetic Reference Panel (DGRP), a collection of approximately 200 fully sequenced inbred lines of *Drosophila melanogaster*^20^, obtained from the Bloomington *Drosophila* Stock Center (BDSC). All flies were reared under standard culture conditions (25 °C, 60–70% humidity, 12 h light-dark cycle). In order to minimize environmental effects on the physiology, we maintained each stock at constant parental density with 30 female flies and 15 male flies over two generations for the metabolic and microbiome profiling experiments. The basal and diet-shift experiments were each performed once. To prevent fluctuations in the food quality, all flies of the respective experiment were provided with food from the identical batch. The impact of vial-to-vial fluctuations was minimized by collecting samples for each DGRP fly line from multiple separate vials.

### Fly food recipes

The standard died (SD) for stock maintenance contains (per 100 mL) 0.5 g agar (Becton Dickinson, 214010), 7.1 g polenta (Verival, Pronurel Bio, 265250), 0.95 g soy flour (Bauck Hof, Amazon.de, B004RG3C0I), 1.68 g yeast (Bruggeman, lieferello.de, 14874413), 4 g treacle (Original Grafschafter Goldsaft, lieferello.de, 10231869), 4.5 g malt extract (Demeter, Amazon.de, B00GU029LW), 0.45 mL propionic acid (Acros Organics, 220130010, CAS 79094) and 1.5 mL nipagin (Sigma-Aldrich, H3647-100G) (1:10 stock solution in 70% Ethanol; Riedel-de Haën, 16202S-1L, CAS 64-17-5). For the experiments with different food conditions, a subset of the DGRP stocks was reared on low sugar diet (LSD) and the adults were discarded after five days of egg-laying from the LSD. After eclosion, freshly hatched adults were transferred to new LSD, high sugar diet (HSD) or SD for five days. The different food types are based on the Bloomington semi-defined medium^21^ with adjustment in the sugar concentration^22^. 100 mL of the basic medium contains 1 g agar, 8 g yeast, 2 g yeast extract (Becton Dickinson, 212750), 2 g peptone (Becton Dickinson, 211677), 200 µl [1 M] MgSO_4_ (Grüssing, 12094), 340 µl [1 M] CaCl_2_ (Sigma-Aldrich, 12074), 600 µL propionic acid, 1.5 mL nipagin and 5.13 g sucrose for LSD or 34.2 g sucrose for HSD (Roth, 4661.1).

### Developmental time

For the quantitative determination of the developmental time, we photographed the fly vials every day at the same time until all examined lines showed eclosed flies. Based on the developmental timing, we arranged the fly lines into five groups.

### Biochemical measurements

For the biochemical assays, unless otherwise described, we collected five late third instar larvae, which left the food and are therefore post-feeding, and eight six day old adult flies (males and females). We washed the larvae in cold PBS to remove the food that might be attached to the outside of the larvae (and dried them on tissue paper). We then snap froze the animals in liquid nitrogen and stored them at −80 °C. For the homogenization, we used 400 µL 0.05% Tween 20 (Sigma-Aldrich, H3647-100G) in 2 mL screw-cap tubes using a Fast Prep FP120 machine (Bio101 Savant). After a heat-inactivation step at 70 °C and short centrifugation (1 min at 2.400 × g), the supernatant was transferred into new 1.5 mL tubes and used for the biochemical assays described below. For each assay, we used three replicates. All measurements are normalized to the number of animals per sample used in the assay.

#### Triglycerides

The TAG content quantification was performed using the Triglycerides Reagent (Thermo Scientific, 981786). The assay is based on the enzymatic cleavage of the fatty acid moieties from the glycerol backbone of mono-, di-, and triacylglycerols and the subsequent quantification of the released glycerol. A detailed description of the method can be found at^23^. Monoacylglycerols are negligible in various insects^24^ and *Drosophila*^23,25^ and only very little diacylglycerol and free glycerol amounts are present, which are negligible in comparison to the triacylglycerol amounts^23,25^. In this study, we actually also quantified free glycerol levels, however, a fluorometric assay was used given the extremely low levels of free glycerol (see below). In brief, the assay was performed by transferring an aliquot of the supernatant of the above mentioned homogenate (25 µL) to a 96 well plate (Sarstedt, 82.1581) followed by a dilution with 25 µL 0.05% Tween 20 (1:2). Samples and standard were incubated at 37 °C with the Infinity reagent and the absorbance was measured at 510 nm. The total amount of TAG was determined using a glycerol standard (Sigma Aldrich, G7793).

#### Glycerol

The glycerol content of the samples was determined using the Glycerol Assay Kit (Sigma-Aldrich, MAK117) according to the manufacturer’s instructions for the fluorometric detection.

#### Glucose and Glycogen

For the quantification of glucose and glycogen content in our samples, we used the GO Assay Reagent (Sigma-Aldrich, GAGO20) and a protocol for determining glucose and glycogen content in flies described in^26^. For the glucose measurement, the female samples were diluted 1:2 (no dilution step for the males and the larvae). For the glycogen measurements, the larval samples were diluted 1:2, the male 1:3 and the female samples 1:6 for the experiment under basal conditions and 1:10 for the diet-switch experiment.

#### Protein

The protein content was measured using the Pierce BCA assay kit (Life Technologies, 23225) according to the manufacturer’s instructions. A standard with defined amounts of bovine serum albumin (BSA) was used to determine the total amount of proteins in the samples.

#### Lactate

For the lactate determination, adult flies and larvae were homogenized in 400 µL PBS. The samples were measured fluorometrically with the Lactate Assay Kit (Biovision, K607-100) following the manufacturer’s instructions. The larval samples were diluted 1:15 and the adult samples 1:10 in PBS.

#### Citrate Synthase Activity

For the determination of the Citrate Synthase activity we used five flies and homogenized them quickly in 100 µL cold PBS. After a short centrifugation step (5 min, 1200 × g, 4 °C), we transferred 20 µL of the samples to a 96 well plate (Sarstedt, 82.1581) and added 200 µL of a reaction mix described in^27^. For the determination of the Citrate Synthase activity, we measured the change in absorbance due to the reduction of 5,5′-Dithiobis(2-nitrobenzoic acid) (DTNB) (Sigma-Aldrich, D8130-1G) at 412 nm and used the following equation (equation 1),

1 $$Citrate\,Synthase\,activity\,(\frac{U}{mL})=\frac{\,({\rm{\Delta }}{A}_{412}/\min )\cdot {V}_{reaction}(mL)\cdot dil}{\varepsilon (m{M}^{-1}\cdot c{m}^{-1})\cdot L(cm)\cdot {V}_{enz}(mL)}$$

where ΔA_412_/min describes the change in absorbance at 412 nm per minute, V_reaction_ is the total reaction volume (0.22 mL), V_enz_ is the volume of the sample (0.02 mL), dil is the dilution factor of the sample and L is the layer thickness (for a 96 well plate approx. 0.63 cm). The attenuation coefficient ε describes the attenuation of the light of a chemical solution based on the path length. The attenuation coefficient of TNB^2^- is 13.6 mM^−1^ cm^−1^.

### Quantification of egg-laying

For the quantification of the fecundity of the four DGRP lines with male metabolic profiles (DGRP lines 324, 380, 732, 786), we used one virgin and two males per vial. We collected virgins and aged them for two days separately from the males to be sure that they were sexually mature. After the two days, we put one virgin together with two males from the same age. After 24 h, we transferred the flies onto new standard diet and counted the eggs. We measured the egg-laying for four days and started with 15 to 20 vials per DGRP line (raw data is given in Supplemental table, sheet C). To be sure that an altered egg-laying rate stems from the female flies, we used wild type male flies (Oregon R). As control lines we used two DGRP lines (362, 714) with “normal” female metabolic profiles. During the experiment, dead males were replaced with back-up flies from the same age and vials with dead females were discarded and excluded from the analysis.

### DNA extraction from whole *Drosophila* flies

For the analysis of the gut bacteria found in *Drosophila*, we extracted genomic DNA using the QIAamp DNA Mini Kit (Qiagen, 51304). For this, eight six day old flies or five late third instar larvae were externally sterilized with 70% ethanol and homogenized in 180 µL ATL buffer, containing 0.5% Reagent DX for foam minimization using an electric pestle (Kimble™ Kontes™ Pellet Pestle, 749540-0000). For additional lysis, we added 20 µL Proteinase K solution to the samples and incubated for 30 min at 56 °C with shaking at 650 rpm. The samples were further lysed by homogenization using glass beads (425–600 µm, Sigma Aldrich, G8772-100G) in a Fast Prep FP120 machine (Bio101 Savant) and afterwards incubated another 60 min at 56 °C. For RNA digestion, we added RNase A (Qiagen, 19101) and incubated the samples for 2 min at room temperature. Next, 200 µL buffer AL were added to the samples and incubated for 30 min at 70 °C. Another 10 min at 95 °C ensured complete lysis of the cells. After cool down, 200 µL ethanol p.a. were added and the samples transferred to the spin column. The washing and elution steps were performed according to manufacturer’s instructions. The samples were afterwards further concentrated by sodium acetate precipitation.

### Next generation sequencing

For sequencing of the *Drosophila melanogaster* gut microbiome, the variable regions of the 16S gene V3 and V4 were amplified using the following primers^28^; 16S Amplicon Forward Primer: 5′ TCGTCGGCAGCGTCAGATGTGTATAAGAGACAGCCTACGGGNGGCWGCAG 3′ and 16S Amplicon Reverse Primer: 5′ GTCTCGTGGGCTCGGAGATGTGTATAAGAGACAGGACTACHVGGGTATCTAATCC 3′; including the Illumina adapter sequences (underlined). Sequencing was performed using the Illumina MiSeq System. Libraries were prepared and sequenced at the Genomics & Transcriptomics Lab of the Heinrich-Heine-University, Duesseldorf using the Illumina 16S metagenomic sequencing library preparation protocol.

All sequence analysis were performed using the MG-RAST pipeline 4.0.2 (released 31.05.2017). Reads were aligned to the SILVA database^29^ and results were processed with R (R core team 2014; version 3.3.1).

### PCR detection of *Wolbachia* in *Drosophila*

In order to screen the flies of the DGRP for an infection with Wolbachia, we used the primers described in^30^. The primers target an intergenic region of the *Wolbachia* genome (A-supergroup repeat motif (ARM)) that can be found in many copies throughout the *Wolbachia* genome in *Drosophila melanogaster*^30^. The PCR was performed as described in Schneider *et al*.^30^.

### Phenotypic correlation analysis

We examined correlations among our measured metabolic traits of the 35 DGRP lines under basal conditions. We performed the correlation analysis in R (R core team 2014; version 3.3.1) and used the spearman rank correlation method to determine the correlation coefficients using our line mean values of the measurements. Additionally, we calculated the P values for each correlation with the AS 89 algorithm^31^.

### Genome wide association studies (GWAS)

To identify single-nucleotide polymorphisms (SNPs) that are associated with metabolic traits we performed genome wide association studies with the help of a web-based analysis tool on the DGRP website (http://dgrp2.gnets.ncsu.edu). We filtered the list providing the top hits among the trait-associated genome variants (provided from the DGRP tool website) for statistical significance with a P < 10^−5^ cut-off. All identified SNPs are listed in the supplemental table (sheet G, H, J). We also counted the unique trait-associated SNPs and classified the SNPs based on the presence or absence of a clear-cut gene association. Additionally, we provide in the supplemental table (sheet I, K) the information of the unique trait-associated genes. Manhattan plots were prepared using the R package “qqman” (R core team 2014; version 3.3.1) and a significance threshold of P < 10^−5^.

### Further data analysis

All analyses were performed using custom scripts written in the R language (R core team 2014; version 3.3.1).

### Data availability

All metabolic measurements and GWAS information are provided in the supplemental table (sheet A to L). The microbiome sequencing data is deposited in the MG-RAST website (https://metagenomics.anl.gov/) and will be made public upon acceptance of the manuscript.

## Results

### The phenotypic plasticity of wild type flies

**Figure 1:**
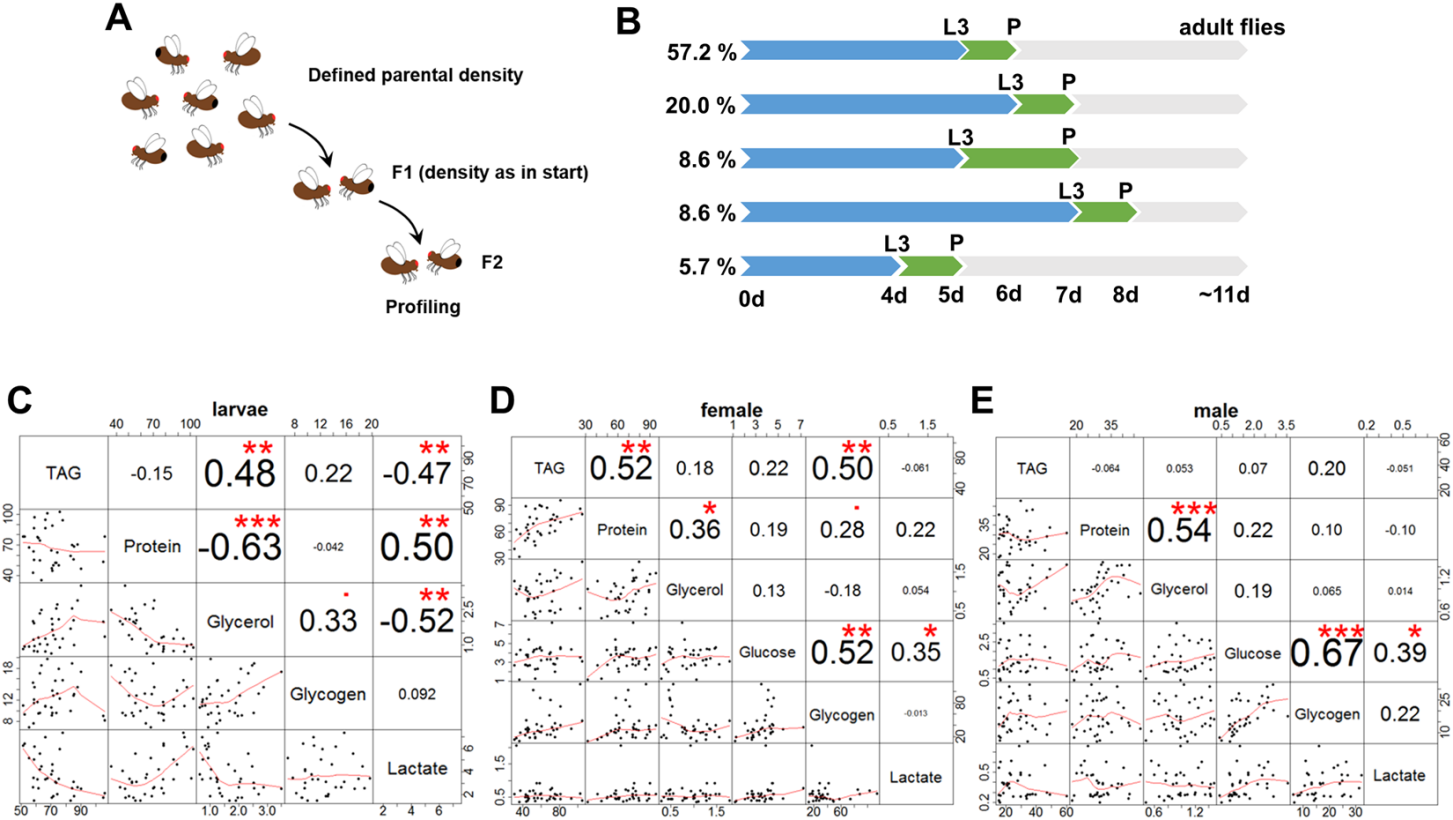
Metabolic profiling of selected DGRP fly lines. (**A**) Experimental outline of the profiling experiment. In order to minimize environmental effects, we kept the population density constant for two generations. The subsequent offspring was metabolically profiled. Additionally, samples were collected for the gut microbiome sequencing experiments. (**B**) The investigated fly lines showed differences in the developmental timing. While the majority of fly lines showed the expected developmental timing, several lines showed differences in either the larval or pupal development. The adult flies hatched almost all at the same time. Late third instar larvae, female and male flies were collected and triacylglycerol (TAG), total protein, free glycerol, lactate, glycogen and glucose were measured. In order to identify linked metabolites, we performed correlation analyses for larvae (**C**), females (**D**) and males (**E**). Profiling experiment following scheme presented in (**A**) performed once. Asterisks in (**C**–**E**) indicate statistical significance levels with: *p < 0.05, **p < 0.01 and ***p < 0.001 based on t-test statistics.

As a proxy for the phenotypic space covering the metabolic repertoire of *Drosophila melanogaster*, we used 35 fly lines of the previously well-characterized *Drosophila melanogaster* Genetic Reference Panel (DGRP), a set of wild caught, inbred fly lines which are fully sequenced^19,20^. As differences in the rearing conditions could have confounded our results, we minimized the effects of population density and associated food and stress inequalities by keeping the parental density constant over two generations (Fig. 1A, see Materials and Methods for detailed explanations). Subsequently, we first investigated the developmental timing of the progeny under standard nutritional conditions. With ten to eleven days, all fly lines showed a comparable timespan needed to complete development. Yet, we noted prominent differences in terms of the time needed to complete larval and pupal development (Fig. 1B). In order to test whether such developmental differences correlate with altered metabolic parameters, we collected late third instar larvae (mixed sex) as well as six day old mated male and female flies. For each sample, we measured the amount of total protein, glucose, glycogen, free glycerol, triacylglycerol (TAG) and lactate. For the adult flies, we additional measured the citrate synthase activity as a proxy for aerobic metabolic activity.

Most metabolic parameters showed prominent differences across the DGRP fly lines (Figs S1 and S2 and Supplemental Table, sheet A). Also in terms of the relative metabolite amounts within the larval, female and male samples considerate differences were evident. Late third instar larvae, for example, store lower amounts of glycogen in comparison to the adult flies (Figs S1 and S2). Glucose levels in late third instar larvae were even below the detection limit in some of the DGRP lines while they were easily quantifiable in the adult samples. Interestingly, the larval lactate amount of some DGRP lines was up to 20-fold higher than in the adult male and female samples. This is potentially caused by a high activity of lactate dehydrogenases (LDH) during larval development in comparison to the pupal and adult stages^32^. LDH reduces pyruvate to lactate under anaerobic conditions and regenerates NAD+ , which is required for glycolysis^32^. Besides the differences between larval and adult stages, a clear-cut sexual dimorphism in terms of the metabolite distribution is evident (Fig. S2). The amounts of triglycerides and glycogen in the adult flies (males and females) were much higher than free glycerol and glucose. Only the activity of citrate synthase (E.C. 2.3.3.1; CS) stays nearly constant in both sexes of all DGRP fly lines. CS catalyzes the initial step in the TCA cycle to convert oxaloacetate and acetyl-coA to citrate. CS activity is therefore a proxy for mitochondrial aerobic capacity^27^. While in pupae of *Drosophila* CS activity is low, there is an increase in adult tissue that corresponds to an increase in metabolic activity^27^, which appears to be under tight control given the low variability we observed.

To learn more about the coupling of metabolic reactions and pathways, we investigated next whether there are informative pairwise-correlations among the metabolite measurements. Intuitively, for example, we expected that a “thrifty-genotype” is marked by the simultaneous accumulation of lipid and carbohydrate stores. At the same time, we also imagined a possible balance between different energy storage metabolites; i.e. that high neutral storage lipid amounts result in low carbohydrate storage levels and vice versa. In order to investigate whether the metabolic profile correlates with differences in the developmental timing, we first performed k-means cluster analysis for the larval metabolic profile, which resulted in the identification of six larval metabolic prototypes (Fig. S3A,B). Subsequent correlation tests with the developmental timing data, however, did not result in clear-cut coupling between metabolism and developmental timing (Fig. S3C). Yet, when we tested for inter-metabolite correlations, we found different statistically significant correlations (Fig. 1C–E and Supplemental Table, sheet B). For example, TAG and glycogen levels in females showed a strong positive correlation (P < 0.002) (Fig. 1D). The lack of this correlation in the male data (Fig. 1E) might suggest that the high energy demand of reproduction asks for a maximum energy storage capacity. Also during the late third instar larval stage, remarkable positive and negative correlations between lactate, triglycerides, protein and glycerol were present (Fig. 1C).

**Figure 2:**
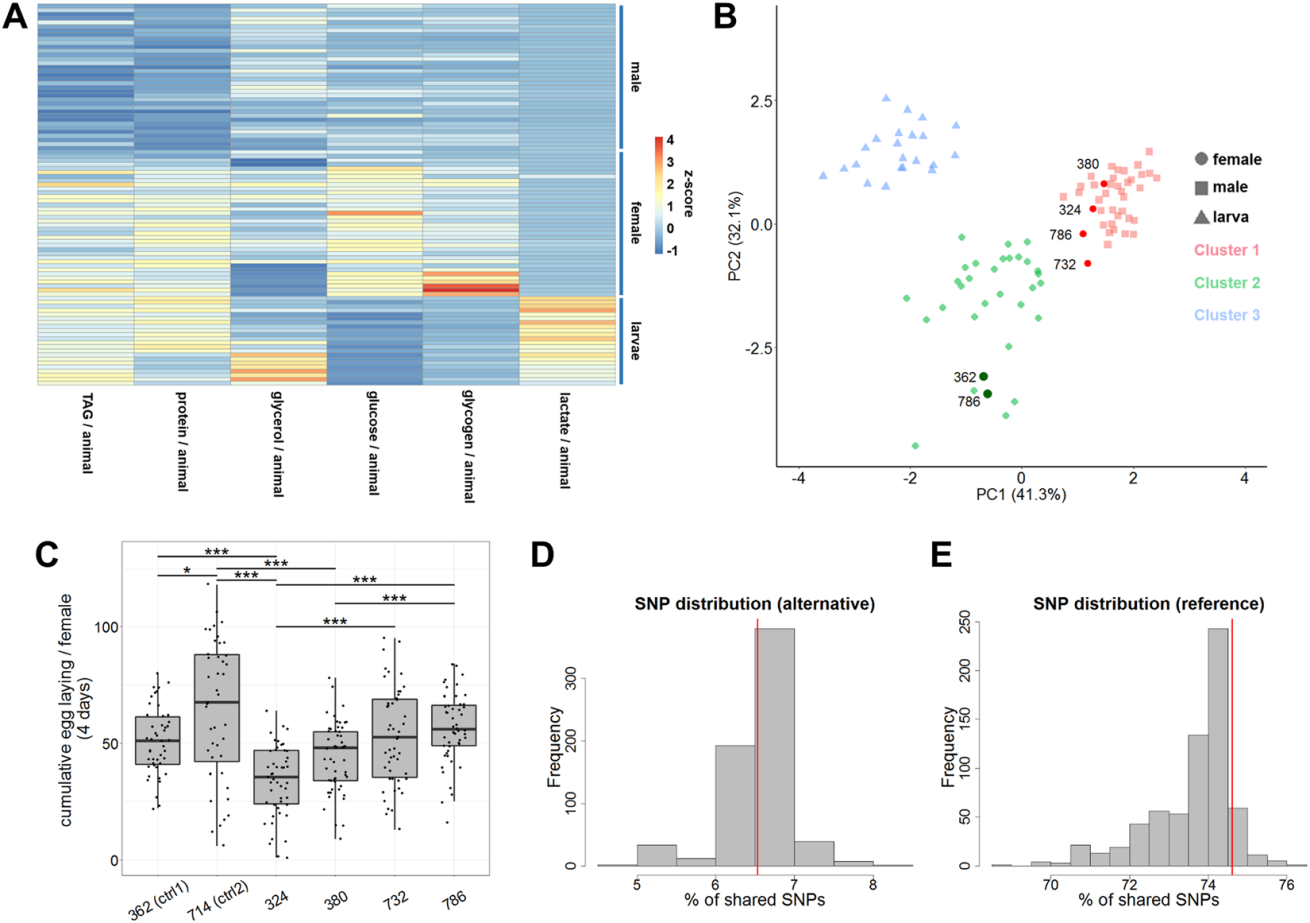
Multiparametric analysis of the metabolic profiling data. (**A**) Heatmap showing the k-means clustered metabolite profiling results. Color coding represents the column-wise z-score normalized metabolite measurements. Each row represents a single sample. Different metabolic cohorts can be identified. (**B**) Principal component analysis of the metabolite profiling experiments shown in (**A**). The data segregates into three clearly-distinguished clusters labeled green, blue or red. The colors almost perfectly align with the samples (larvae, females and males). However, cluster 1 (males) contains four data points, which are of female origin. (**C**) The four DGRP lines, where female samples were assigned to the male cluster, were tested for a putative fertility phenotype. The box plots show the averaged results of three biologically independent egg-laying quantification experiments (complete experimental data shown in Supplemental table, sheet **C**). Significant differences based on an ANOVA analysis and Bonferroni corrected post-hoc testing are labeled by the asterisks. Significance levels: p < 0.05 *; p < 0.001 ***. In order to identify possible SNPs associated with the masculinized metabolic profile of the four DGRP lines we investigated the per cent of shared alternative (**D**) or reference (**E**) alleles. In brief, we calculated the amount of shared alleles between any two of the 35 DGRP fly lines used and plotted the results as a histogram. Subsequently, we compared the resulting distribution with the value obtained for the four masculinized DGRP lines (red line).

We subsequently investigated whether our metabolic data would also be sufficient to identify distinct “metabotypes”, which we define as cohorts of fly lines sharing a certain metabolic profile. K-means clustering indeed revealed for the larval, female and male metabolite profiles three clear-cut metabolic clusters (Fig. 2A). The metabolite profiles were also sufficiently different to result in three clearly separable clusters in a principle component analysis matching the larval, male and female samples (Fig. 2B). The protein and TAG levels mainly contributed to the PC1, whereas PC2 was mostly driven by glycogen and glucose levels (Fig. S3D). When we analyzed the PCA in greater detail, we intriguingly found that four of the six day old female samples (DGRP lines 324, 380, 732, 786) were more similar to the male than the female samples based on their metabolic profile (mainly due to lower TAG, glycogen, glucose and protein amounts; cf. Supplemental Table, sheet A). Based on this classification, we defined them as so-called “virago” lines. We subsequently tested whether this “male-like metabolite profile” might be indicative of a masculinization. Given that in humans a masculinization is accompanied by high testosterone titers and a decreased fertility and reproductive success^33,34^, we tested the virago DGRP fly lines for an altered egg-laying rate and capacity in comparison to DGRP lines with a clear-cut female metabolic profile (DGRP lines 362, 714). We mated the female flies of the different DGRP lines to males of the unrelated *Drosophila* wild type laboratory strain Oregon R for 24 h (for detailed explanations see Material & Methods) and measured the mean cumulative number of eggs per female from three independent biological replicates (Fig. 2C and Supplemental table, sheet C). Two of the four virago lines (324, 380) indeed showed a significantly decreased egg-laying rate (Fig. 2C and Supplemental table, sheet C). Our results nicely align with the results of the PCA where the virago lines with reduced egg-laying rate are most distant from the female-type cluster and lie directly in the male metabolic cluster (Fig. 2B). Given that the DGRP set of fly lines is fully sequenced^19,20^, we asked next whether we could identify genetic differences, which possibly account for the metabolic and/or reproductive phenotype of the virago fly lines (only for 324, 380). For this purpose, we searched the single nucleotide polymorphism (SNP) information available for the DGRP lines^19^ for an enrichment or depletion of SNPs in the virago lines as compared to the other DGRP fly lines. Because we did not know how big the overlap between the different lines was, we decided to perform all pairwise comparisons and look at the similarity distribution (see Materials and Methods). For the alternative alleles, the per cent of shared SNPs of the viragos was not different from the most-frequently expected result of the non-virago fly lines (6.53% versus 6.52%; Fig. 2D). For the reference alleles, the level of conservation among the virago lines was slightly higher than the most-frequently expected result (74.61% versus 73.59%; Fig. 2E). Given the lack of a clear-cut enrichment of reference or alternative alleles in the virago lines, we searched for reference or alternative alleles that only appear in the two virago lines (324, 380). Indeed, we detected 22 reference and 418 shared alternative alleles (Supplemental table; sheet D) that might have an influence on the metabolic profile and/or the fecundity of the flies. However, none of the detected allelic variants are known to impact metabolic or reproductive phenotypes, thus asking for further studies.

### The metabolic response to changing nutritional conditions varies among fly strains

**Figure 3:**
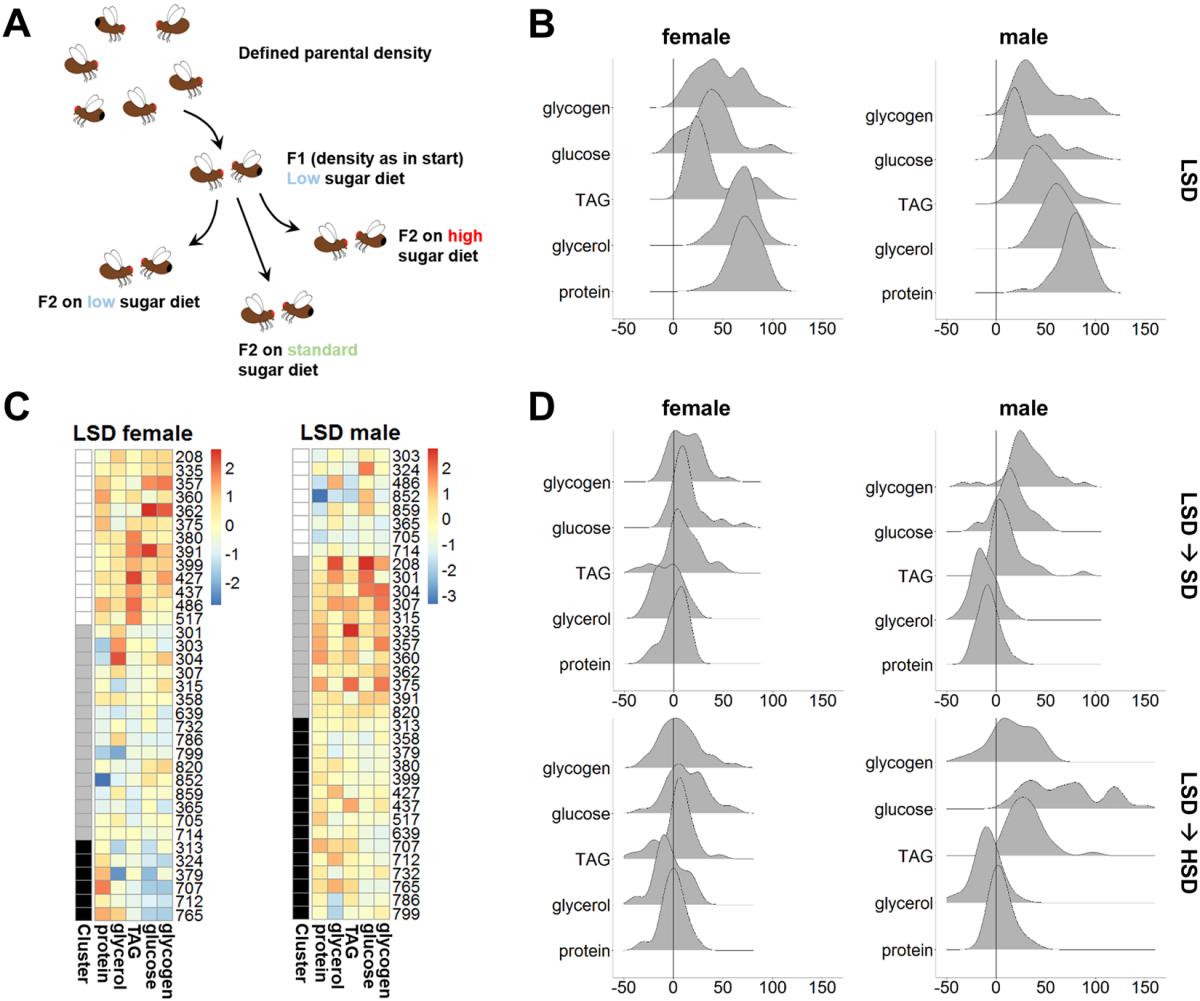
Metabolic response to a changing diet. (**A**) Experimental outline. As shown in Fig. 1A, we kept different DGRP fly lines under constant parental density for two generations. This time, the animals were kept on a low sugar diet. The hatched animals subsequently remained on a low sugar diet, or were shifted to richer food (standard diet or high sugar diet). (**B**) At the age of six days, the animals were sacrificed to measure the total protein, glycerol, triacylglycerol (TAG), glucose and glycogen content by targeted assays. For all measurements  values were per cent normalized among the different fly lines. The distributions of metabolic values for the five measurements for the female and male samples are shown. (**C**) Heatmaps for the clustered female and male samples. (**D**) Density plots showing the metabolite amount differences following the shifts from low sugar diet (LSD) to standard diet (SD) or high sugar diet (HSD), respectively. Profiling data as schematized in (**A**) was performed once.

The flexible response of individuals to varying nutritional conditions is a well-known phenomenon. Some people, for example, react to high caloric food with less weight gain as compared to others, despite of a similar overall lifestyle. In order to investigate whether flies also show such a plastic response, and to investigate the genetic contribution to this phenomenon, we extended our metabolic profiling experiments to flies challenged with varying sugar amounts in the food (Fig. 3A). Given that we were mainly interested in the identification of variations in the propensity to gain energy stores, we decided to let the animals develop on a diet with reduced sugar content (low sugar diet; LSD), before transferring the flies to different diets with increased sugar amounts. As in the basal metabolic profiling experiments, we kept the flies prior to the actual experiment at a defined parental density on standard food for two generations. Subsequently, we allowed the animals to lay eggs on a diet with reduced sugar content (low sugar diet; LSD). After hatching, the animals matured for five more days on the LSD or we transferred them for the five day maturation to a standard, complex cornmeal-molasses diet (standard diet; SD) or a high sugar diet (HSD; see Materials and Methods for diet compositions) before measuring the same metabolites as we did for the basal experiments. The SD and HSD foods both have a higher sugar content as compared to the LSD (see Materials and Methods). Thus, a switch from the LSD to each of the other diets should result in increased energy storage levels. Indeed, the glucose amounts averaged across the measurements of all DGRP fly lines of both sexes showed this expected increase (Fig. S4A). For TAG and glycogen levels, however, we observed a sex-specific response to the diet change (Fig. S4B,C). Male TAG levels increased more strongly following the switch to the high sugar diets. For glycogen, it was the opposite way and the female flies showed a more prominent response to the increased sugar content. The averaged total protein (Fig. S4D) and glycerol (Fig. S4E) content of the flies of both sexes was independent from the sugar content of the food.

When we investigated the variation of the metabolic parameters of flies raised and matured on the LSD, we decided to per cent normalize the metabolite measurements and investigated the distribution of metabolite amount frequencies (Fig. 3B, for all measurements see Supplemental Table, sheets E and F). Our results largely corresponded to the findings of the basal metabolite profiling experiment (Figs S1 and S2), where the DGRP lines showed for most of our measurements variable amounts. An exception are e.g. the total protein amounts per fly, which only show modest variation. This limited variability corresponds to a Gaussian-like distribution (Fig. 3B). The female TAG levels of most DGRP lines, however, were low which results in a prominent peak in the distribution with another peak demonstrating the presence of some fly lines where females had high TAG storage levels. For the males, in contrast, the distribution of TAG amounts across the DGRP lines is broad and almost bell-shaped. Glycogen and glucose levels also showed high variation across the tested DGRP fly lines (Fig. 3B). As for the basal metabolic profiling experiments, clustering analysis resulted in the identification of different metabotypes among the female and male fly samples (Fig. 3C) characterized by e.g. either relatively low or high TAG storage levels.

The main focus of the diet shift experiment was of course to investigate whether all DGRP lines would show comparable metabolite increases following the diet shift. In order to answer this question, we used the per cent normalized metabolite measurements of the LSD samples and calculated the relative per cent increase or decrease of the given measurement following the diet shift. Positive per cent values thus reflect an increase, whereas negative per cent values reflect a decrease of the given measurement. In order to visualize the changes we used either dumbbell-plots (Fig. S5) or investigated again the distribution of metabolite changes (Fig. 3D). Following the transfer to the SD, most fly lines increased their glucose, glycogen and TAG levels. As expected, the protein levels stayed mostly constant. Intriguingly, however, a number of fly lines showed decreased levels of free glycerol following the diet shift (Fig. 3D). When the flies were shifted from the LSD to the HSD, the glucose levels showed the largest increase across the collection of DGRP fly lines. Similarly, the glycogen and TAG levels also mostly increased. Again only the levels of free glycerol showed a reduction following the altered sugar content of the food. On the level of individual DGRP fly lines, the changes varied indeed prominently (Fig. S5) showing both increases as well as decreases in certain metabolites such as TAG (Fig. S5A,B) or glycerol (Fig. S5C,D). Interestingly, the energy storage level changes appeared to be independent of the initial storage compound amounts. For example, the TAG levels of the male DGRP fly lines 705 and 427 are both within the lower third of the investigated fly lines and displayed very different responses with a 95% and 14% increase following the shift to the high sugar diet (Fig. S5B).

### The genetic contribution to metabolic diversity

**Figure 4:**
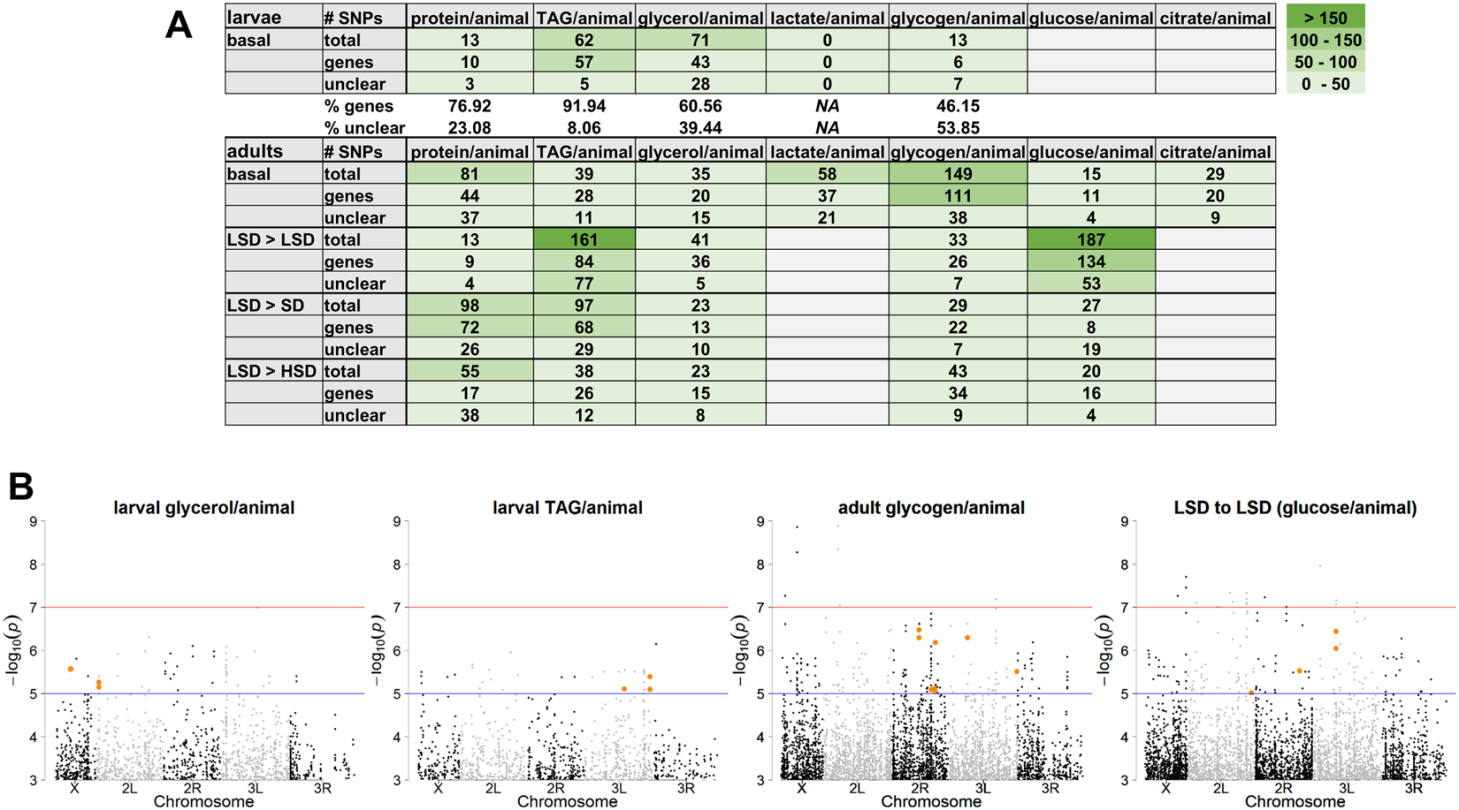
Genome-wide association studies (GWAS) to identify phenotype-genotype mappings. (**A**) Table providing the number of total, gene-associated and unclear mapped single nucleotide positions (SNPs) associated with the traits measured in the basal and diet shift metabolic profiling experiments. Color code represents the number of SNPs. (**B**) Exemplary manhattan plots for SNP associations with larval glycerol, larval triacylglycerol, adult glycogen and adult glucose (grown on low sugar diet) levels. Significance thresholds are 10^−5^ (blue) and 10^−7^ (red line). Orange color highlights genes with a known function in metabolism or metabolic regulation such as *slimfast*, *melted*, *smooth*, *short neuropeptide F* and its receptor. For details see text.

In order to search for genome variances associated with the metabolic plasticity observed under basal and metabolically challenged conditions, we performed genome-wide association studies (GWAS) for the late third instar larvae and six day old adult fly data (males and females) using standard procedures^19^. The DGRP set of fly lines is a proven tool for mapping phenotype-genotype associations^35–37^. At a p-value significance threshold of 1 × 10^−5^ we identified a number of SNPs that are associated with the different metabolic traits of the larvae and adult flies (Fig. 4A and supplemental table; sheets G, H, J). Intriguingly, the number of detected SNPs showed a high variability across the different traits (Fig. 4A). For example, while the variation of larval glycerol and lactate levels was comparable (Fig. S1), we did not detect a single SNP associated with lactate levels, whereas we identified 71 glycerol-associated SNPs (Fig. 4A). Figure 4B depicts manhattan plots for selected trait-associated SNP associations. Besides SNPs targeting intergenic regions as well as genes, which were previously not associated with metabolic function, we detected several SNPs associated with genes encoding previously described metabolic regulators (all SNP-associated genes are provided in Supplemental Table, sheets I and K). For example, the late third instar larvae TAG data were associated with SNPs linked to *scylla (scyl*) and *short neuropeptide F receptor (sNPFR)*. Scylla is a member of the target of rapamycin signaling pathway, which affects TAG storage levels^38^. *sNPFR* is involved in the satiety regulation and foraging behavior^39^. Thus, a connection to energy storage regulation is very likely. GWAS analysis of the larval glycerol measurements could be linked to SNPs associated with the genes *neural lazarillo*, *lysophosphatidylcholine acyltransferase (LPCAT)* and *CG1986*. The gene *neural lazarillo* is a known metabolic regulator linking stress response to metabolism^40^. For *LPCAT* and the annotated gene *CG1986* less information is available^41^. Yet, *LPCAT* is presumably involved in phospholipid synthesis and mutations of *LPCAT* homologs have been shown to affect lipid storage levels and lipid storage organelle morphology^42^. The *CG1986* gene is an annotated lipase and therefore potentially involved in metabolic responses. For the adult flies analyzed under basal conditions, we identified *smooth* (correlated with glycerol, glycogen), *slimfast* (associated with glycogen), *stretchin-Mlck* (associated with glycerol, lactate), *trehalase* (associated with glycerol) and *retinal homeobox* (associated with glycogen). The gene *smooth (sm)* is known to play a role in food intake regulation and survival^43^. *Slimfast (slif)* is included in the lipid metabolic process and monitors the amino-acid concentration in the fat bodies^44^. Ugrankar and colleagues used physiological and genetic interrogations to find genes that are involved in the glucose metabolism in flies^45^. Through this “glucome” screen they identified amongst others *stretchin-Mlck (Strn-Mlck)* and *retinal homeobox (Rx*).

For the diet shift experiment, we also identified several genes known to play a role in metabolic processes such as *fat body protein 1 (fbp1)*, *melted (melt)*, the *glucose transporter 1(Glut1)* or the *LDL receptor protein 1 (Lrp)*. The gene *melt*, for example, is involved in the regulation of lipid metabolism of flies^46^. The mutant *melt∆1* shows a reduction in body size (10%) and triglyceride levels (40%) in comparison to the control animals^46^. The *fbp1* gene is expressed only in late third instar larvae and is directly controlled by ecdysteroids. The gene functions as receptor for Larval Serum Protein 1 (LSP1) and enables the import of hexamerin into the fat body^47,48^.

When we analyzed the 565 SNPs detected under basal and 878 SNPs detected under diet shift conditions for an overlap, we detected only six identical SNPs showing up under both conditions (see Supplemental table; highlighted in sheets H and J). On the gene level, we detected 256 genes under basal and 377 trait-associated genes under diet shift conditions with an overlap of 32 genes (see Supplemental table; highlighted in sheets I and K). While for the majority of the overlapping genes the link to metabolic regulation is not yet clear, we also identified two important metabolic key players. First, the *leucokinin receptor (Lkr)* is involved in meal size regulation^49^. Second, we identified SNPs in the enzyme *trehalase (Treh)*, which not only breaks down hemolymph trehalose to make the sugar moieties accessible for metabolism, but which was also demonstrated to be important for the adaptation to varying nutritional conditions^50^.

### The microbiome – metabolism interaction

**Figure 5:**
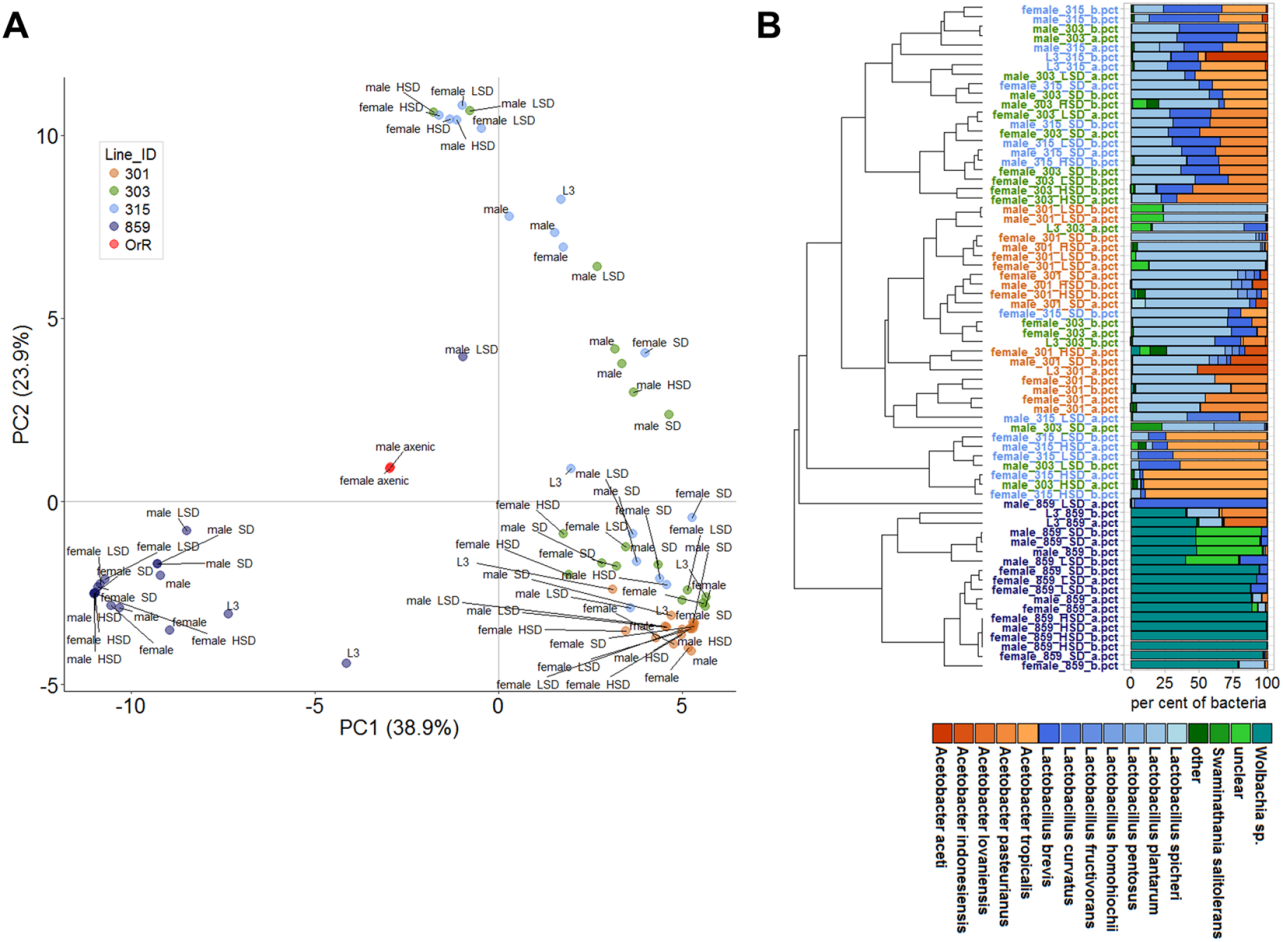
Microbiome composition of four selected DGRP lines in response to varying metabolic compositions. (**A**) Principal component analysis of the microbial compositions of animals raised on a standard diet (late third instar larvae, six day old females and males) or animals raised on a low sugar diet or shifted from a low sugar diet to a standard or high sugar diet (six day old female or male flies). (**B**) Hierarchical clustering of stacked bar plots of the sequencing results shown in (**A**). Each mentioned bacterium had to be among the top 95% most abundant bacteria in at least one of the 72 samples. All samples were collected in parallel to the samples obtained for the basal metabolic profiling experiment (Fig. 1) and the diet-shift metabolic profiling experiment (Fig. 3). All sequencing experiments were done with duplicate samples.

Aside of the genetic variation, the bacteria present in the gut – the so-called gut microbiome – profoundly affect the metabolism, physiology and development of many if not all multicellular organisms^51–57^. Yet, the functional link between given bacterial species and certain metabolic traits is not clear. We thus utilized our metabolic profiling data to investigate this question by testing the hypothesis that certain metabolic traits correlate with microbiome constituent abundancies. Given that we tested for correlations across independent experiments and different nutritional conditions, we expected only robust correlations to survive. As a starting point, we selected from our collection of DGRP fly lines four representative strains (301, 303, 315 and 859) showing different metabolic profiles in the basal experiment (Fig. S6A,B) and altered metabolite responses to the diet shift (Fig. S7). Importantly, the flies were collected in parallel to the metabolic profiling experiments, thus eliminating an uncoupling of the metabolic and microbiome sequencing data. For replicate samples of larval, female and male representatives of the four fly lines we sequenced the V3 and V4 region of the 16S rRNA gene. As negative controls, we additionally sequenced one female and one male wild type fly sample (Oregon R), grown in the absence of a gut microbiome (“axenic”). For the axenic samples, the sequencing resulted in much smaller read numbers, which predominantly mapped to bacteria found in the human microflora of the skin and mouth. For the other samples, *Acetobacter* and *Lactobacilli* species dominated, as expected for laboratory-reared *Drosophila melanogaster* samples^56^. PCA analyses also showed that the axenic samples clearly separated from the conventionally reared, metabolic profiling samples (Fig. 5A). One of the DGRP lines (859) formed a distinct cluster separated from one large cluster where the samples of the remaining three fly lines were intermingled. Additionally three smaller clusters with a mixture of the DGRP lines 303 and 315 were visible. A clear-cut separation of developmental stages or diet treatments was not visible. Hierarchical clustering based on the top 95% of the microbial species largely confirmed the PCA results (Fig. 5B). For the clustering, we removed the axenic samples, as we wanted to focus on the species composition of the conventionally reared samples. Interestingly, we noted only smaller differences among the major microbiome constituents within the different samples. The microbiome of DRGP line 301 predominantly constituted of *Lactobacillus plantarum* and only smaller amounts of *Acetobacter* species. The DGRP lines 303 and 315 displayed varying amounts of different *Acetobacter* in addition to *Lactobacillus* species, which separated into smaller clusters with both lines intermingled depending on the amount of the main bacterial species. As already noted in the PCA analysis, we did not observe prominent changes on the microbiome composition following incubation on the different diets (shift from LSD to HSD or LSD to SD) and the clusters predominantly contain samples of the same fly line.

The only major difference notable was the high abundance of *Wolbachia pipientis* in the DGRP line 859. *Wolbachia* is not a member of the *Drosophila* gut microbiome, but acts as an endosymbiont, which profoundly impacts the host^58^. We subsequently tested all our DGRP lines for *Wolbachia* infection using a specific PCR amplification strategy^30^. 15 of the 35 lines were tested *Wolbachia* positive. Based on the availability of our metabolic profiling data, we tested whether significant correlations exist with the presence of *Wolbachia*. This, however, was not the case (data not shown). On the level of the most abundant species, the hierarchical clustering results in the same main cohorts of related fly lines, which we also identified by PCA (Fig. 5A). Our experiments neither revealed a clear-cut microbiome composition change in response to the different diets nor the different sexes or developmental stage (larvae versus six day old adults).

### Correlations among metabolites and microbiome species

**Figure 6:**
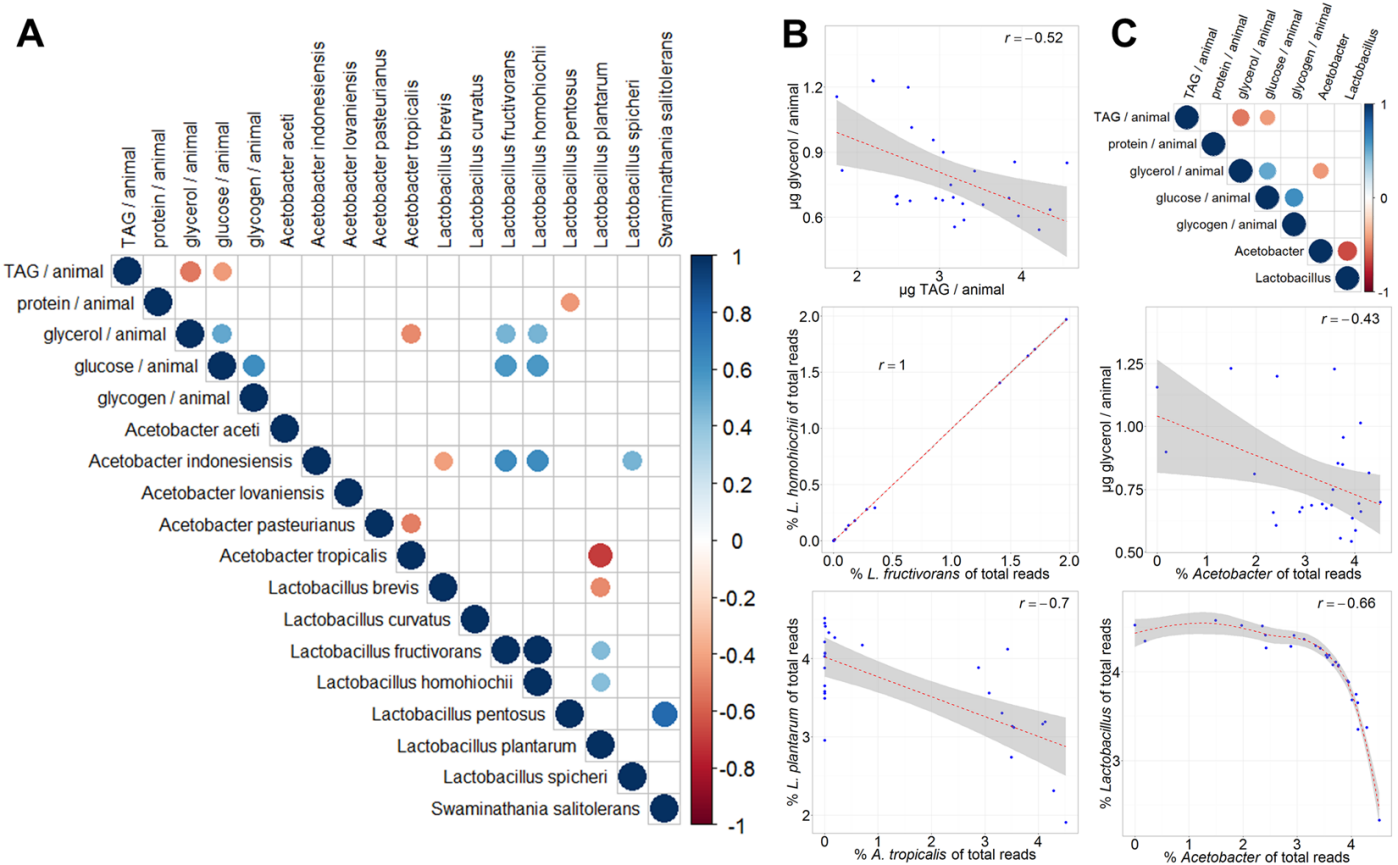
Correlation between metabolic parameters and gut microbiome bacterial abundancies. All data are log transformed. The DGRP line 859 and the *Wolbachia* species were excluded due to fact that *Wolbachia* is not a natural member of the gut microbiome. (**A**) Correlation matrix of significant correlations (p < 0.05) between the indicated parameters and the bacterial abundancies on species level. The color code and size of the circles are proportional to the r correlation coefficient and the p-value. (**B**) Scatter plots of selected correlations from the correlation matrix shown in (**A**). The linear approximation and the confidence interval as well as the r values are shown. (**C**) Correlation matrix of significant correlations (p < 0.05) between the indicated parameters and the bacterial abundancies on genus level. Scatter plots of selected correlations from the correlation matrix shown above.

An interesting and still open question is how tight the link between gut bacteria and the physiology of the host is. We contribute to answer this question by correlating our metabolic measurements with the quantifications of the most abundant bacterial species of the gut (bacterial species had to be among the top 95% in at least one of the samples). Given that a large portion of the sequencing reads of the DGRP line 859 were assigned to *Wolbachia* – which is not a gut endosymbiont – we excluded these samples from the correlation analysis, which we limited to significant correlations with p-values smaller than 0.05 (see Materials and Methods). Among the metabolite measurements we detected several significant correlations as, for example, glycerol and triacylglycerol (r = −0.52, p < 5.3 × 10^−3^) (Fig. 6A,B), which also correlated in the larval measurements performed under ad libitum fed conditions (Fig. 1C). Also the abundancies of different bacterial species showed significant correlations. Examples include *Lactobacillus plantarum* and *Acetobacter tropicalis* (r = −0.7, p < 4.9 × 10^−5^) or *L*. *homohiochii* and *L*. *fructivorans* (r = 1, p < 6.4 × 10^−45^) (Fig. 6A,B and Fig. S8B). Surprisingly, however, correlations between metabolites and selected bacterial species abundancies were not as strong. We therefore tested next whether the metabolites would rather match up with bacterial abundancies on the genera level. When we correlated the two main bacterial genera *Lactobacillus* and *Acetobacter* with the metabolites (Fig. 6C), we indeed could detect a negative correlation between free glycerol and *Acetobacter* (r = −0.43, p < 2.5 × 10^−2^). The genera *Lactobacillus* and *Acetobacter* additionally showed a nonlinear correlation (r = −0.66, p < 1.97 × 10^−4^). Our results therefore suggest that the linkage between metabolite levels and certain microbial species is weaker than expected.

## Discussion

Here, we investigated the interplay between the genetic variation and dietary changes on the metabolic phenotype and the microbiome composition. As a baseline, we performed metabolic measurements of different DGRP lines reared under ad libitum conditions on a standard diet (Figs S1 and S2). As previously reported^35^, we detected a considerable phenotypic variability in respect to energy storage levels. Based on our measurements, it is possible to classify groups of fly lines within the core set of DGRP fly lines as “very skinny”, “normal” and “fat”. A possible source of this phenotypic variability could be differences in developmental timing. Yet, we were not able to find clear-cut correlations between metabolic phenotypes and the varying developmental paces (Fig. S3A–C) suggesting that the metabolic differences were rather based on e.g. an altered metabolic activity as compared to extended or shortened developmental phases and thus altered feeding periods.

Our set of measurements, of course, was limited. In the future, it will be interesting to expand our studies by measuring additional metabolites. The disaccharide trehalose, for example, is the major sugar transport form within the hemolymph of insects^59^ and thus would be interesting to analyze. Further, metabolomics methodologies are more recently also applied to *Drosophila*^60–63^ and allow for a more global survey of metabolites, which will allow even more fine-grained analyses and deeper insights. Using metabolomics, for example, the differential metabolic repertoire of female and male flies as suggested by gene expression analyses^64^ could be confirmed^65,66^. One of the studies revealed that females show a higher proportion of acylated amino acids, which might be associated with the microbiome based on their enrichment in the gut^65^. Tissue specificity and a more fine-grained analysis of the interaction between the metabolic profile, the gut microbiome composition and the impact of environmental factors thus will be highly informative. Again, metabolomics should provide a rapid means to achieve this goal, as it was previously used to investigate the metabolome throughout the lifecycle of *Drosophila*^67^ and in different tissues of the fly^68^, although the absolute quantifications, which we performed with the targeted assays, are still difficult to achieve using mass spectrometry based metabolomics methods^69^.

We asked next whether there are correlations between different metabolite abundancies in the late third instar larvae, six day old female and male flies (Fig. 1C–E). Given that glucose and glycerol are subunits of glycogen and triglycerides, respectively, we supposed clear-cut correlations. Glucose and glycogen indeed showed a positive correlation in the male and female adult flies and TAG and glycerol levels positively correlated in the larvae. On top of these expected correlations, we also found interesting new ones such as the strong correlation between lactate and TAG, glycerol, or protein, respectively, in the larvae. This finding might be indicative of the at least partial hypoxic lifestyle of larvae, which could explain the generally much higher lactate amounts during this developmental stage. While direct correlations are clearly the most informative, also the lack of correlation is potentially meaningful. One example is the positive correlation between TAG and glycerol in the larvae, which is not present in the male and female adult flies. This finding may be indicative of the high lipogenic activity of the larvae, where lipid levels increase tremendously thus asking for a high amount of available free glycerol to enable the esterification of fatty acids to form TAG. In adults, storage lipid levels are under *ad libitum* conditions less dynamic, which potentially explains the lack of correlation.

Our limited metabolic profiling data was already sufficient to clearly separate the larval, male and female samples in a PCA (Fig. 2A). This result clearly supports sex-biased metabolic differences as previously reported^70–72^. To our surprise, however, we also found four female fly lines showing a male metabolic profile (Fig. 2A). As masculinization is often accompanied by a reduced fecundity, we investigated the egg-laying capacity. For two of the four lines, we indeed saw a significantly reduced rate of egg-laying (Fig. 2C). Given that the other two fly lines did not show a different fecundity, the metabolic differences observed by us are likely not only based on the increased metabolic demand of the egg production process. While we investigated the genome sequence^19,20^ for variances shared or enriched in the virago lines, we only identified nucleotide polymorphisms mapping to genes lacking a clear-cut link to metabolism or reproduction regulation. Further investigations are thus needed to understand the influence of a given metabolic profile on the reproductive success and vice versa but this is beyond the scope of our study.

The question why some individuals are able to cope with a high sugar diet much better than others in terms of the weight gain is still mostly unanswered. Here, we introduce the fly as a model system to investigate this question, and identified among the DGRP lines prominently different metabolic responses to diets with variable sugar content (Fig. 3 and Figs S4, S5).

Our genome-wide association studies revealed a number of genes carrying genetic variations significantly associated with the profiling measurements performed under basal and diet switch conditions. Luckily, not all of the genetic variants mapped to intergenic regions or introns, but also to coding regions. For the beginning, we concentrated on the latter ones given that it is often close to impossible to find a functional link between an intergenic SNP and a given trait. Among the genes with metabolite-associated SNPs we identified several proof-of-principle examples such as *stretchin-MLCK*^45^ or *retinal homeobox*^45,73^, where targeted experiments previously demonstrated a metabolic phentoype associated with a gene perturbation. Intriguingly, the overlap of genetic variants associated with metabolic traits identified under basal and metabolically challenged conditions was low with only six identical SNPs and 32 SNP-associated genes showing up in both conditions. This finding suggests that under nutritional challenging and basal conditions a different set of genes is important. Different previous studies from various groups also used the DRGP set of flies to assay for metabolism-genotype interactions^35,37,45^ or investigated the heritability and inter-population differences in lipid profiles of inbred *Drosophila* isolates from various geographical locations^74^. While the comparison of absolute metabolite amounts is still difficult - as discussed for example for triglycerides based on alterations in the homogenization procedure, number of flies per sample or food differences etc.^23^ – previous reports showed a similar spread of metabolite measurements as we observed across the DGRP fly lines^35^. The studies of Jumbo-Lucioni *et al*.^35^ and Ugrankar *et al*.^45^ indeed identified a number of metabolite-associated gene variants shared by our study (see Supplemental table, sheet L), suggesting robustness of the experimental procedure.

The symbiosis between the host and its gut microbiome has a deep impact on the host’s metabolic state^75^ and there are big hopes that targeted microbiome alterations will be available for personalized medicine therapies^76,77^. Yet, it is still under debate how fast microbiome compositions change and how stringent the directionality between a certain metabolic phenotype and a given microbial community is. Here, we wanted to analyze the correlation between a certain gut microbiome and a metabolic phenotype of the host. In order to minimize the differences between the metabolic measurements and the microbiome profiling, we collected samples in parallel and 16S sequenced the gut microbiome of four DGRP fly lines showing distinct metabolic profiles under basal conditions (Fig. S6) and which showed metabolite changes following the diet switch conditions (Fig. S7). We detected the typical bacteria found in lab-reared *Drosophila melanogaster* and saw a more or less DGRP-line specific microbiome composition (Fig. 5). When we investigated the correlation between metabolite amounts and bacterial species abundancies, we detected only weak correlations across the different fly lines and experiments (Fig. 6 and Fig. S8). While we did see a change in the metabolite levels (Fig. S7), the microbiome composition did not show clear changes following the dietary switch (Fig. 5), which potentially broke the linkage. Why the diet change did not impact the microbiome compositions at a larger scale is further discussed. Yet, a different timing (shorter or longer time frame) for the diet change might have shown a bigger response and thus potentially a more clear linkage. When we investigated the microbiome – metabolite correlation only for the basal experiment, we indeed found additional significant correlations (Fig. S8B) as for example between TAG and *Lactobacillus brevis* or glucose and *L*. *fructivorans* amounts (Fig. S8C). Stronger correlations were visible between individual species of the gut microbiome, such as a negative correlation of *L*. *plantarum* and *A*. *tropicalis* (Fig. 6B), which might be based on conflicting optimal growth conditions. *Lactobacilli*, for example, are microaerophilic whereas *Acetobacteraceae* prefer an acidic and aerobic environment^78^. The strong, non-linear negative correlation between *Lactobacilli* and *Acetobacter* on the genus level (Fig. 6C) further supports such a mutual exclusion. The environment should at least in part also affect the physicochemical properties of the gut and therefore, the diet should have an impact on the gut composition. An interesting result of our microbiome profiling experiments was, however, the lack of prominent compositional changes following the diet switches. How fast the microbiome composition changes in response to environmental changes is in the light of a projected use of targeted microbiome alterations as a means of personalized medicine^76^ an important question. Currently, the data is not clear how fast the microbiome composition changes. Many publications emphasized the impact of the diet consumed by the host on the gut microbiome composition^79–81^. Also in humans, a change of diet to either a strict animal- or plant-based type apparently led to significant microbiome alterations already 24 hours after the diet change^82^. A situation more similar to our experiments is a mouse study, where the animals were subjected to a diet shift from normal to high sugar diet for two weeks, which also resulted in major changes in the gut microbiome composition^83,84^. As mammals harbor resident gut microbiome species, changes in the microbiome composition appear more difficult as compared to flies, where the microbiome is supposed to be subject to constant replenishment causing the microbiome composition to follow external factors, such as the diet^85,86^. In fact, in *Drosophila* the parental animals inoculate their offspring with a certain microbiome composition via the feces, which potentially indicates that there is no need and evolutionary pressure to maintain a resident and stable gut microbiome^87^. Indeed, none of our diet shifts resulted in a clearly altered microbiome composition whereas the metabolic profiles were affected by the diet switch (Fig. S7). Thus, the tested dietary conditions were either not different enough or the diet shifts did not last long enough to provoke a change of the microbial composition on the food or within the guts of the flies. As an alternative, the alterations could have only affected microbiome constituents absent in our flies or parameters, which were not monitored by us, such as total microbial load. The shift on a high fat diet for three days, for example, did only result in a moderate change of abundance of *L*. *plantarum*^88^. Overall bacterial load and abundance of *Commensalibacter intestini* – which was absent in our flies – was significantly increased^88^. Rearing *Drosophila melanogaster* constantly on a high sugar diet resulted in late larval samples in the appearance of the conditional pathogen *Gluconobacter morbifer*, which we also did not detect in our studies^89^. Further supporting information that extended time periods might be needed to induce diet-derived microbiome alterations comes from a study working with *Drosophila suzukii*, where only a diet shift over the course of more than eight generations led to a diet-based diversification of the present acetic acid bacteria^80^. The relatively stable gut microbiome composition and potentially long time frame needed to affect the gut composition, however, might also indicate that the fly also harbors persistent gut bacteria, which are not easily altered by environmental factors.

## Electronic supplementary material

Supplementary Figures and Captions

Supplemental Table
