# Supplementary material for: The impact of genome variation and diet on the metabolic phenotype and microbiome composition of Drosophila melanogaster
Source: Sci Rep. 2018 Apr 18;8:6215. doi: 10.1038/s41598-018-24542-5 (PMC5906449; doi:10.1038/s41598-018-24542-5)
Supplement: Supplementary file 1 — Supplementary Figures and Captions [file 41598_2018_24542_MOESM1_ESM.pdf]

Supplemental data for the manuscript

The impact of genome variation and diet on the metabolic phenotype and microbiome composition of Drosophila melanogaster

Authored by: Lisa Jehrke, Fiona A. Stewart, Andrea Droste and Mathias Beller

The Supplemental data comprises one Excel Workbook with all primary data and eight figures and the associated legends.

Fig. S1: Metabolic measurements of late third instar larvae under basal conditions. Triglyceride (TAG), glycerol, glycogen and lactate, measurements are shown. The data represent mean values ± standard deviation for triplicate samples. The metabolic measurements are normalized to the number of animals per sample (n=5) and ordered from lowest to highest value. The full dataset can be found in Supplemental Table, sheet A.

Fig. S2: Metabolic measurements of six day old adult flies (males and females) under basal conditions. Triglyceride, glycerol, glucose, glycogen, lactate and citrate synthase activity are shown. The data represent mean values ± standard deviation for triplicate (for citrate synthase activity only unique) samples. The metabolic measurements are normalized to the number of animals per sample (n=8) and ordered from lowest to highest value of the female flies. The full dataset can be found in Supplemental Table, sheet A.

Fig. S3: Identification of sub-metabotypes within the larval metabolic cohort and loading plot of the Principal Component Analysis shown in Figure 2B. (A) K-means analysis of the larval metabolic data. (B) Heatmap showing the results from k-means clustering in (A) with six

metabotypes. Color coding represents the column-wise z-score normalized metabolite measurements. Each row represents a fly line from the DGRP subset and the columns represent the metabolic measurements normalized per animal. (C) Bar plot representation showing the interconnecting between the six sub-metabotypes (M1 to M6) of the larvae and the developmental groups (D1 to D5) of the DGRP flies. The representation shows the per cent values of fly lines with a specific developmental timing that group in a sub-metabotype of the larvae. (D) Loading plot of the PCA shown in Fig. 2B. The loading plot provides information concerning the contribution of the different measurements performed to the principal components 1 and 2.

Fig. S4: Box plots summarizing the protein and metabolite measurements of the six day old adult flies (female and male), which were either kept constantly on a low sugar diet or shifted from a low sugar diet to a standard, or a high sugar diet. Averaged data points from the triplicate measurements available for glucose (A), TAG (B), glycogen (C), total protein (D), and (E) glycerol measurements from all 35 female (light grey) and male (dark grey) DGRP fly lines. All data were normalized to the number of animals per sample. The data refer to the scheme shown in Figure 3A. The full dataset can be found in Supplemental Table, sheet E.

Fig. S5: DGRP line-specific response to the diet switch conditions. The dumbbell plots depict the responses of the 35 DGRP lines (males and females) to the diet shift from LSD (blue) to HSD (orange) in different metabolic traits. (A) TAG changes from female flies, (B) TAG changes from male flies, (C) glycerol changes from female flies and (D) glycerol changes from male flies. The values are normalized to the highest LSD value of the respective metabolite. The data relate to scheme shown in Figure 3A. The full dataset can be found in Supplemental Table, sheet F.

Fig. S6: Metabolic phenotype of DGRP lines 301, 303, 315, and 859 used for the microbiome sequencing experiments. (A) Principle component analysis of the z-score normalized metabolic measurements. (B) Radar plot depicting the z-score normalized metabolic measurements for the four DGRP fly lines. The color code is provided in the legend. Both the PCA as well as the radar plot demonstrates a high diversification of the four fly lines based on their respective profiles.

Fig. S7: Radar plots depicting the metabolic changes following the diet switch of the DGRP lines 301, 303, 315, and 859 used for the microbiome sequencing experiments. Results for each line are shown in a separate radar plot. All fly lines showed metabolic changes in response to the dietary shift from the low sugar diet to either the standard or high sugar diet. Numerical code: 1 – TAG (LSD to SD) male; 2 – TAG (LSD to HSD) male; 3 – glycerol (LSD to SD) male; 4 – glycerol (LSD to HSD) male; 5 – glycogen (LSD to SD) male; 6 – glycogen (LSD to HSD) male; 7 – glucose (LSD to SD) male; 8 – glucose (LSD to HSD) male; 9 – TAG (LSD to SD) female; 10 – TAG (LSD to HSD) female; 11 – glycerol (LSD to SD) female; 12 – glycerol (LSD to HSD) female; 13 – glycogen (LSD to SD) female; 14 – glycogen (LSD to HSD) female; 15 – glucose (LSD to SD) female; 16 – glucose (LSD to HSD) female.

Fig. S8: Correlation between metabolic parameters and the gut microbiome. (A) Scatter plots of selected correlations from the correlation matrix shown in Fig. 6A. The linear approximation and the confidence interval as well as the r values are shown. (B) Correlation matrix of significant correlations ($p<0.05$) between the indicated metabolic parameters measured under basal nutritional conditions and their corresponding microbiome species abundancies. The color code and size of the circles are proportional to the r correlation coefficient and the p-value. (C) Scatter plots of selected correlations from the correlation

matrix shown in (B). The linear approximation and the confidence interval as well as the r values are shown.

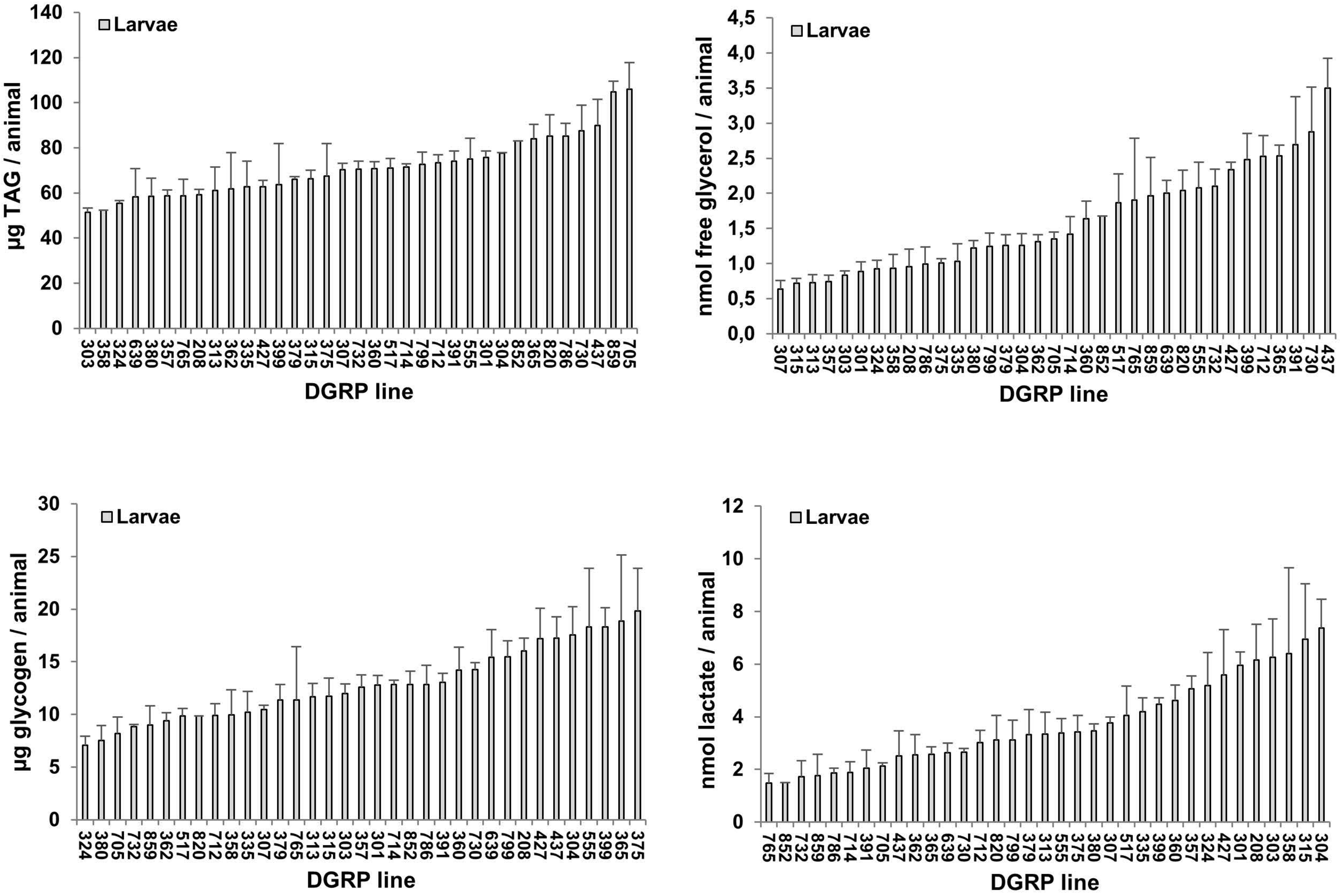
Larvae
μg TAG / animal
140
120
100
80
60
40
20
0
303 358 324 639 380 357 765 208 313 362 335 427 399 379 315 375 307 732 360 517 714 799 712 391 555 301 304 852 365 820 786 730 437 859 705
DGRP line
Larvae
nmol free glycerol / animal
4,5
4,0
3,5
3,0
2,5
2,0
1,5
1,0
0,5
0,0
307 315 313 357 303 301 324 358 208 786 375 335 380 799 379 304 362 705 714 360 852 517 765 859 639 820 555 732 427 399 712 365 391 730 437
DGRP line
Larvae
μg glycogen / animal
30
25
20
15
10
5
0
324 380 705 732 859 362 517 820 712 358 335 307 379 765 313 315 303 357 301 714 852 786 391 360 730 639 799 208 427 437 304 555 399 365 375
DGRP line
Larvae
nmol lactate / animal
12
10
8
6
4
2
0
765 852 732 859 786 714 391 705 437 362 365 639 730 712 820 799 379 313 555 375 380 307 517 335 399 360 357 324 427 301 208 303 358 315 304
DGRP line

Fig. S1

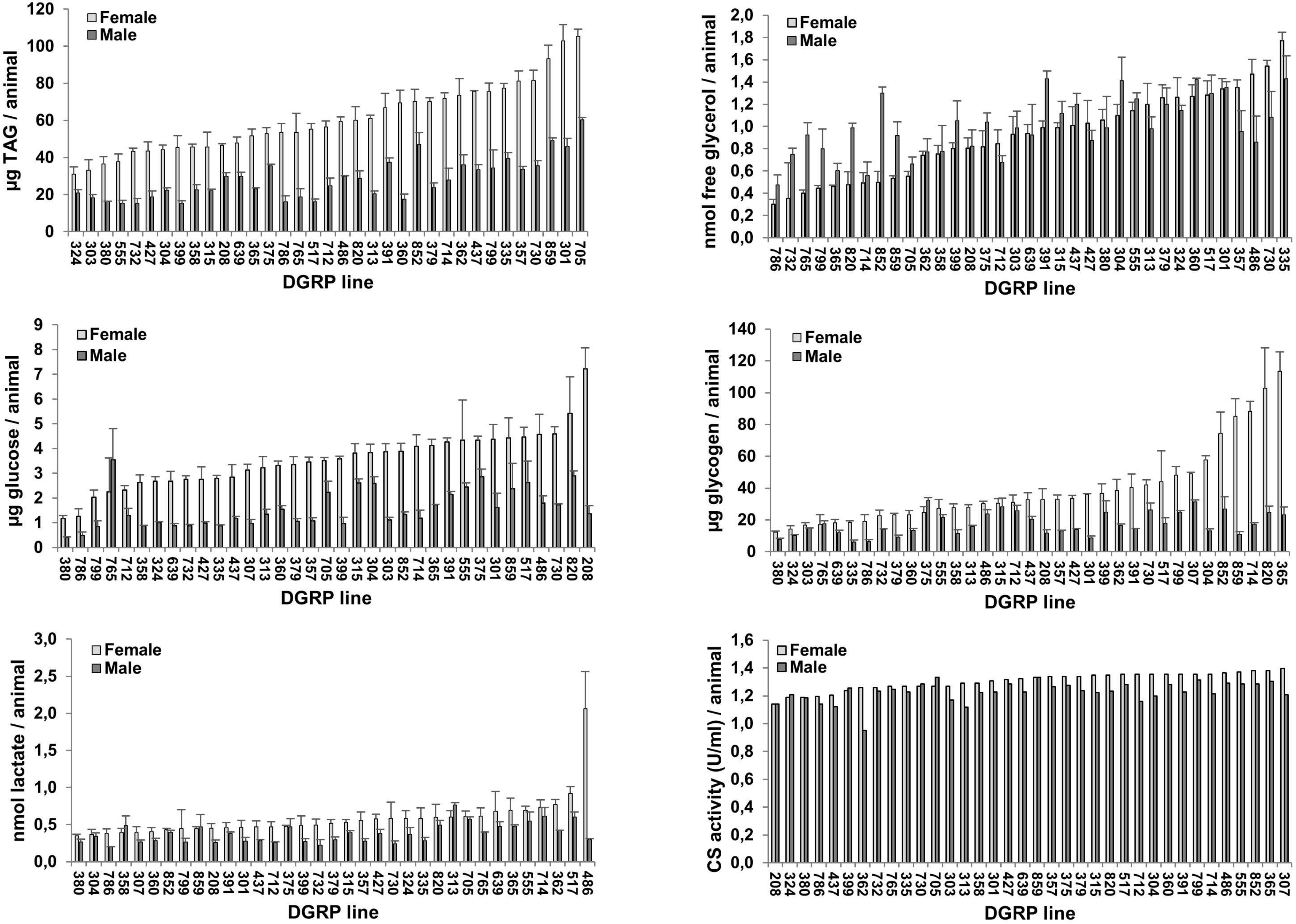
Female
Male
μg TAG / animal
0 20 40 60 80 100 120
324 303 380 555 732 427 304 399 358 315 208 639 365 375 786 765 517 712 486 820 313 391 360 852 379 714 362 437 799 335 357 730 859 301 705
DGRP line
Female
Male
nmol free glycerol / animal
0,0 0,2 0,4 0,6 0,8 1,0 1,2 1,4 1,6 1,8 2,0
786 732 765 799 365 820 714 852 859 705 362 358 399 208 375 712 303 639 391 315 437 427 380 304 555 313 379 324 360 517 301 357 486 730 335
DGRP line
Female
Male
μg glucose / animal
0 1 2 3 4 5 6 7 8 9
380 786 799 765 712 358 324 639 732 427 335 437 307 313 360 379 357 705 399 315 304 303 852 714 365 391 555 375 301 859 517 486 730 820 208
DGRP line
Female
Male
μg glycogen / animal
0 20 40 60 80 100 120 140
380 324 303 765 639 335 786 732 379 360 375 555 358 313 486 315 712 437 208 357 427 301 399 362 391 730 517 799 307 304 852 859 714 820 365
DGRP line
Female
Male
nmol lactate / animal
0,0 0,5 1,0 1,5 2,0 2,5 3,0
380 304 786 358 307 360 852 799 859 208 391 301 437 712 375 399 732 379 315 357 427 730 324 335 820 313 705 765 639 365 555 714 362 517 486
DGRP line
Female
Male
CS activity (U/ml) / animal
0,0 0,2 0,4 0,6 0,8 1,0 1,2 1,4 1,6
208 324 380 786 437 399 362 732 765 335 730 705 303 313 358 301 427 639 859 357 375 379 315 820 517 712 304 360 391 799 714 486 555 852 365 307
DGRP line

Fig. S2

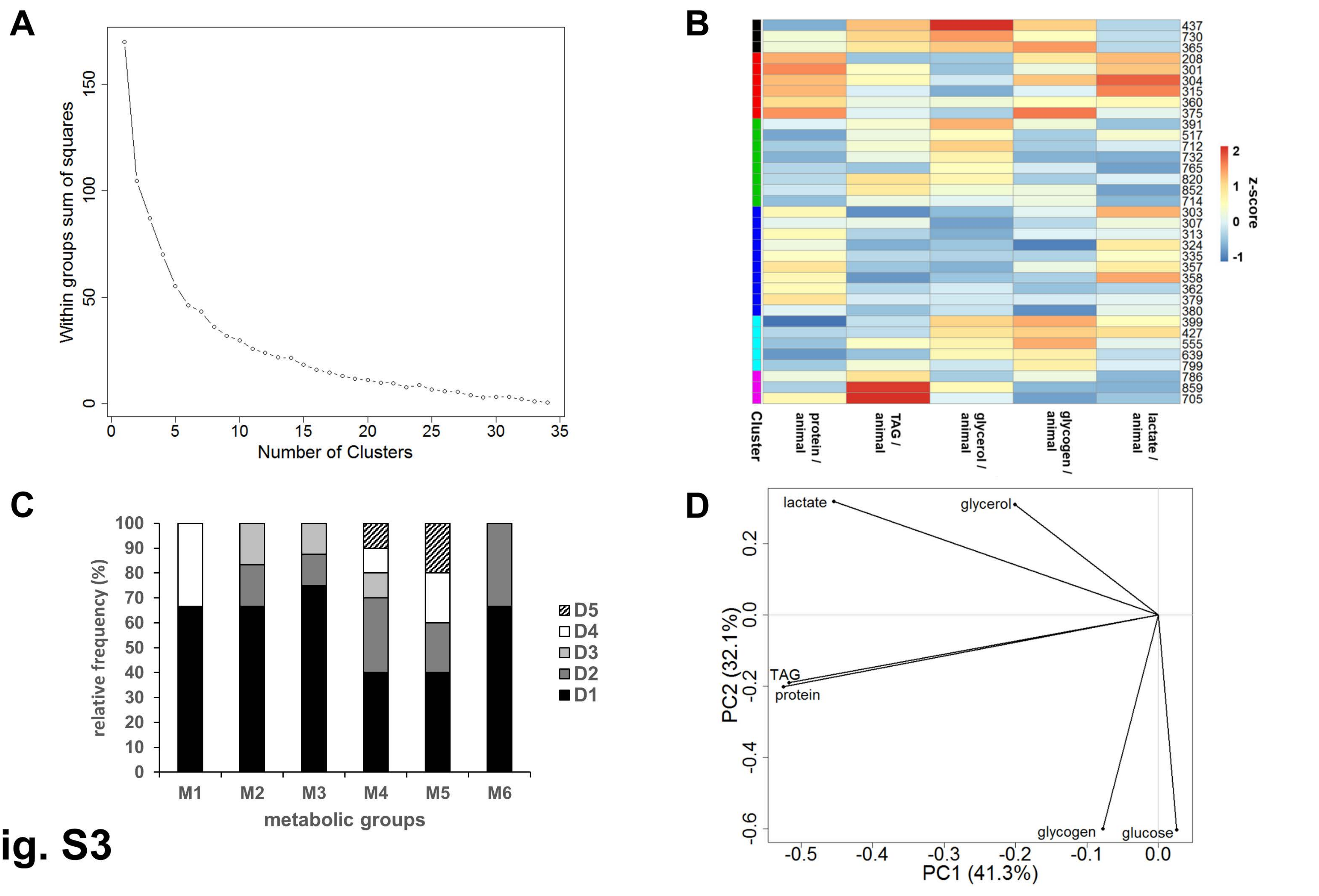
A
Within groups sum of squares
Number of Clusters
B
Cluster
protein / animal
TAG / animal
glycerol / animal
glycogen / animal
lactate / animal
z-score
C
relative frequency (%)
metabolic groups
M1
M2
M3
M4
M5
M6
D5
D4
D3
D2
D1
D
lactate
glycerol
TAG
protein
glycogen
glucose
PC2 (32.1%)
PC1 (41.3%)

Fig. S3

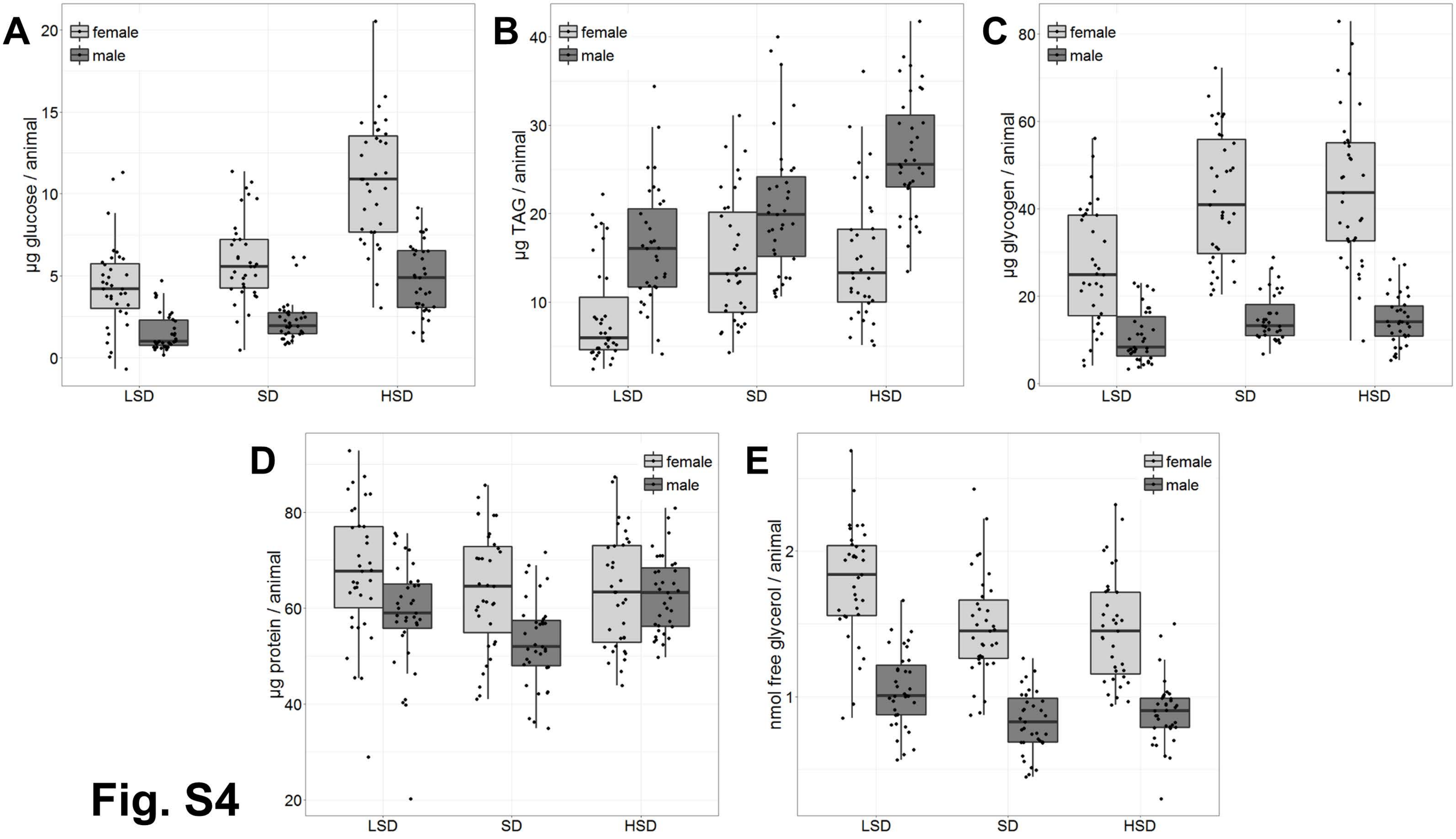
A
B
C
D
E
female
male
µg glucose / animal
µg TAG / animal
µg glycogen / animal
µg protein / animal
nmol free glycerol / animal
LSD
SD
HSD

Fig. S4

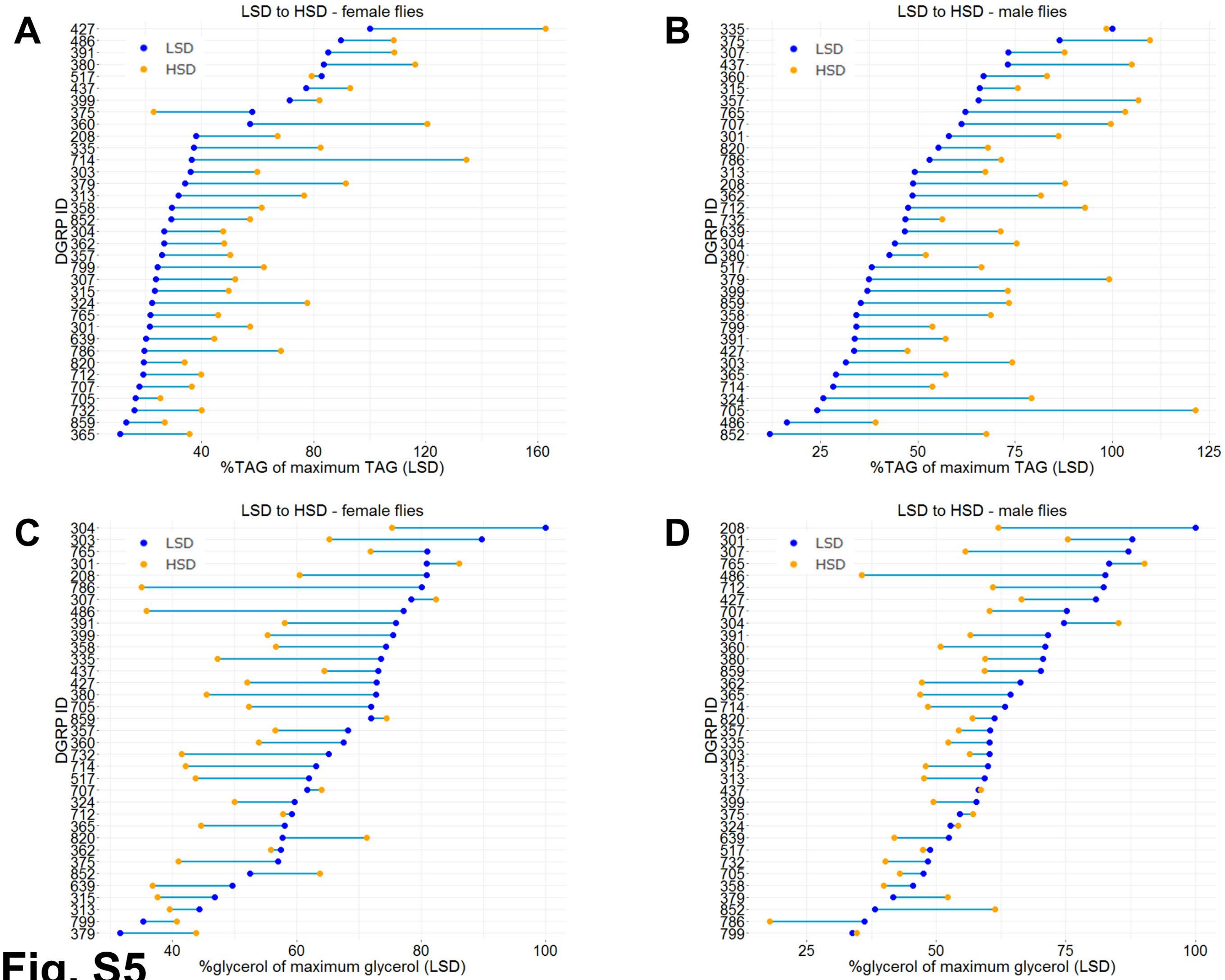
A
LSD to HSD - female flies
LSD
HSD
DGRP ID
427
486
391
380
517
437
399
375
360
208
335
714
303
379
313
358
852
304
362
357
799
307
315
324
765
301
639
786
820
712
707
705
732
859
365
40
80
120
160
%TAG of maximum TAG (LSD)
B
LSD to HSD - male flies
LSD
HSD
DGRP ID
335
375
307
437
360
315
357
765
707
301
820
786
313
208
362
712
732
639
304
380
517
379
399
859
358
799
391
427
303
365
714
324
705
486
852
25
50
75
100
125
%TAG of maximum TAG (LSD)
C
LSD to HSD - female flies
LSD
HSD
DGRP ID
304
303
765
301
208
786
307
486
391
399
358
335
437
427
380
705
859
357
360
732
714
517
707
324
712
365
820
362
375
852
639
315
313
799
379
40
60
80
100
%glycerol of maximum glycerol (LSD)
D
LSD to HSD - male flies
LSD
HSD
DGRP ID
208
301
307
765
486
712
427
707
304
391
360
380
859
362
365
714
820
357
335
303
315
313
437
399
375
324
639
517
732
705
358
379
852
786
799
25
50
75
100
%glycerol of maximum glycerol (LSD)

Fig. S5

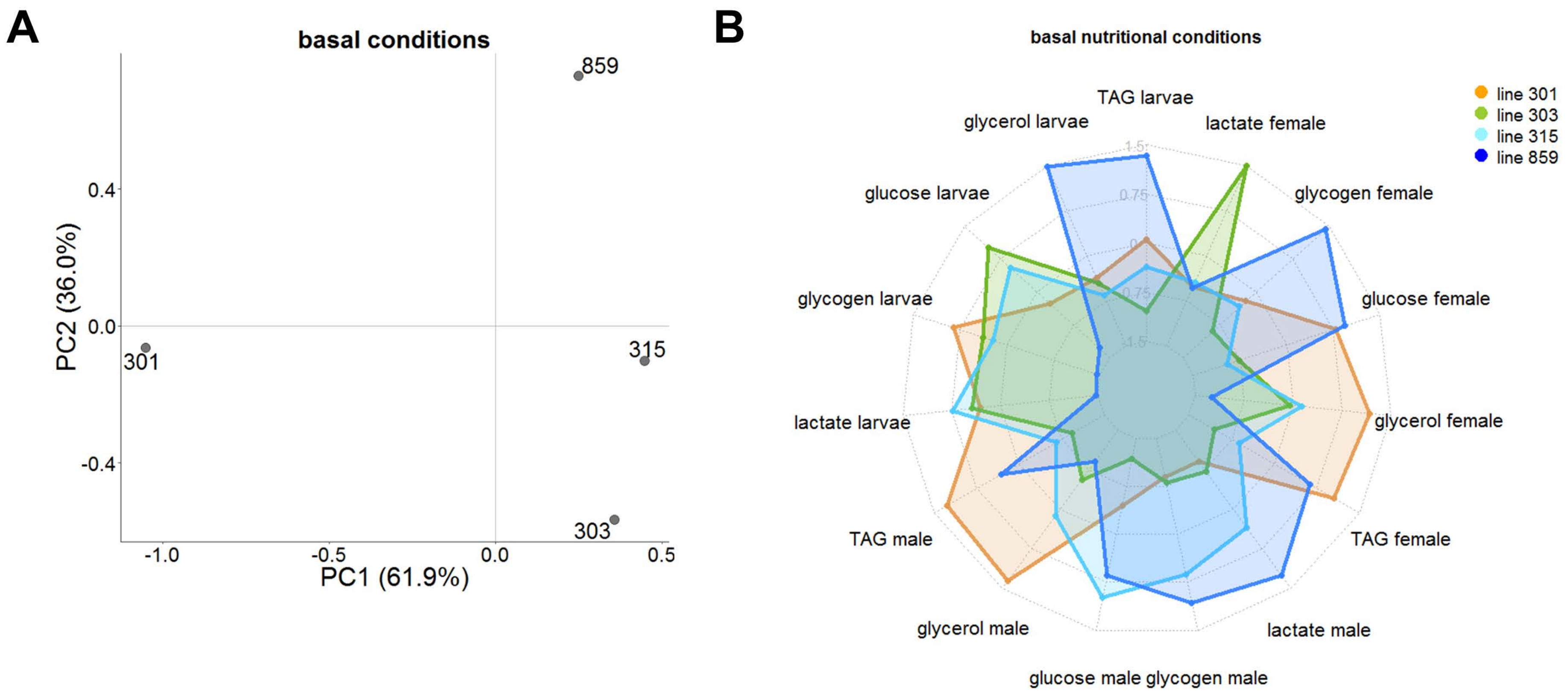
A
basal conditions
859
315
301
303
PC2 (36.0%)
PC1 (61.9%)
0.4
0.0
-0.4
-1.0
-0.5
0.0
0.5
B
basal nutritional conditions
line 301
line 303
line 315
line 859
TAG larvae
lactate female
glycogen female
glucose female
glycerol female
TAG female
lactate male
glucose male glycogen male
glycerol male
TAG male
lactate larvae
glycogen larvae
glucose larvae
glycerol larvae

Fig. S6

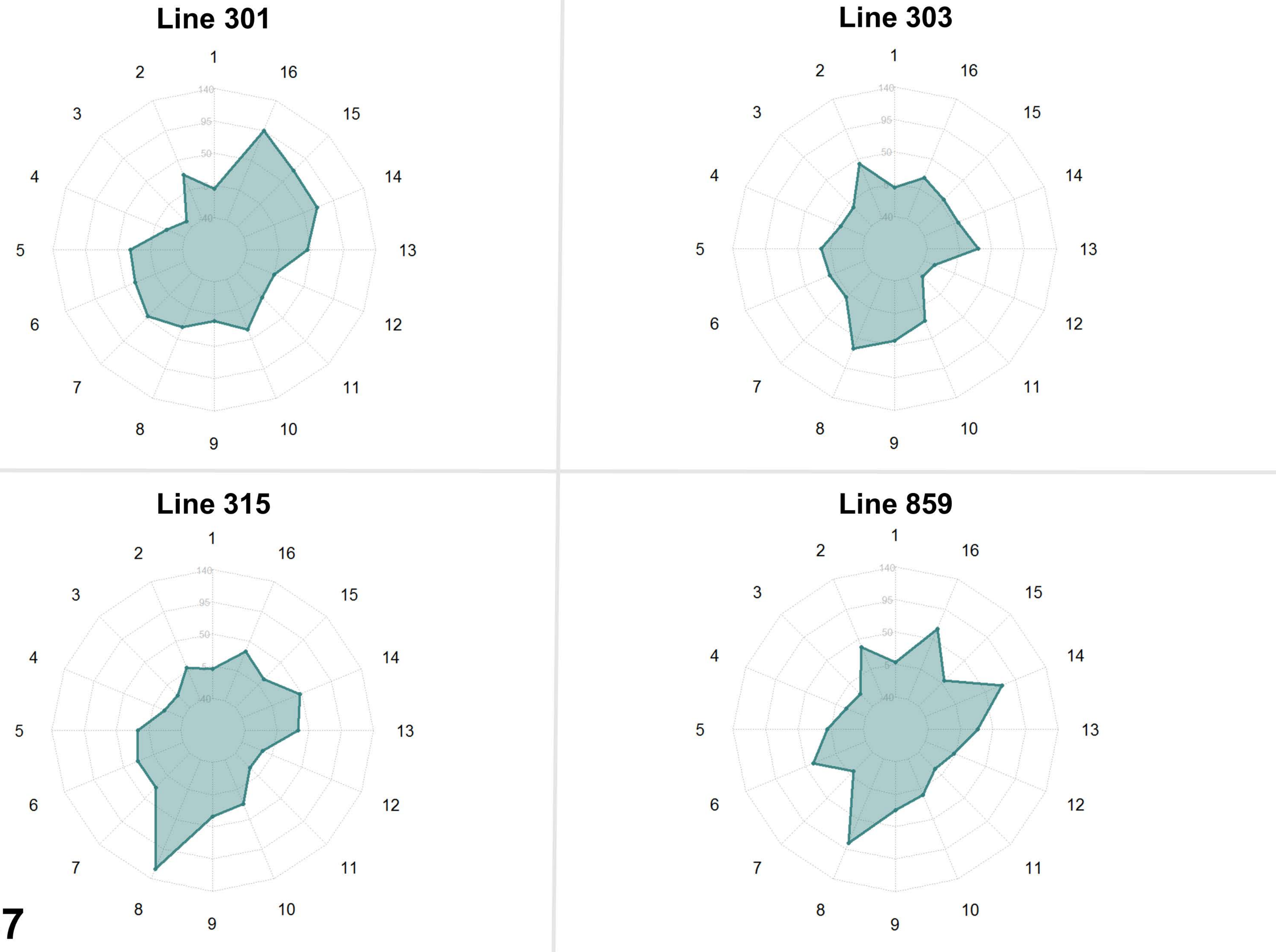
Line 301
Line 303
Line 315
Line 859
1
2
3
4
5
6
7
8
9
10
11
12
13
14
15
16
140
95
50
5
-40

Fig. S7

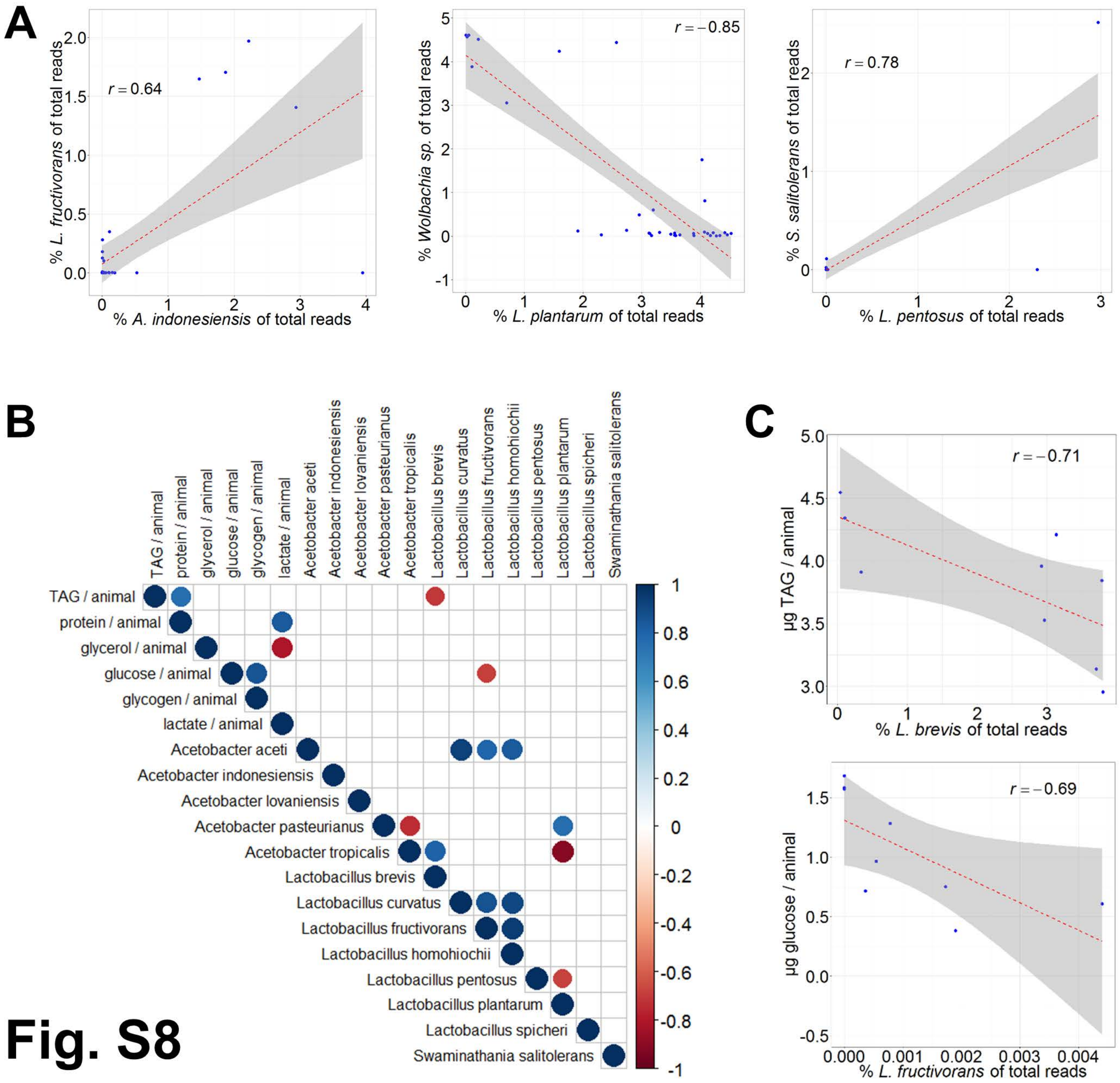
A
r = 0.64
% L. fructivorans of total reads
% A. indonesiensis of total reads
r = −0.85
% Wolbachia sp. of total reads
% L. plantarum of total reads
r = 0.78
% S. salitolerans of total reads
% L. pentosus of total reads
B
TAG / animal
protein / animal
glycerol / animal
glucose / animal
glycogen / animal
lactate / animal
Acetobacter aceti
Acetobacter indonesiensis
Acetobacter lovaniensis
Acetobacter pasteurianus
Acetobacter tropicalis
Lactobacillus brevis
Lactobacillus curvatus
Lactobacillus fructivorans
Lactobacillus homohiochii
Lactobacillus pentosus
Lactobacillus plantarum
Lactobacillus spicheri
Swaminathania salitolerans
C
r = −0.71
µg TAG / animal
% L. brevis of total reads
r = −0.69
µg glucose / animal
% L. fructivorans of total reads

Fig. S8
